# Devonian to Carboniferous continental-scale carbonate turnover in Western Laurentia (North America): upwelling or climate cooling?

**DOI:** 10.1007/s10347-022-00653-4

**Published:** 2022-07-25

**Authors:** Makram Hedhli, Keith Dewing, Benoit Beauchamp, Stephen E. Grasby, Rudi Meyer

**Affiliations:** 1 Geological Survey of Canada, 3303-33rd Street NW, Calgary, AB T2L 2A7 Canada; 2 Department of Geoscience, University of Calgary, 2500 University Drive NW, Calgary, AB T2N 1N4 Canada

**Keywords:** Laurentia, Carbonate, Devonian, Carboniferous, Upwelling, Climate

## Abstract

The Devonian to Carboniferous (DC) transition coincided with a green-to-ice house climatic shift, anoxia, disappearance of lower latitude carbonate banks, and turnover from warm-to-cool water carbonate factories. In western Laurentia, the switch to carbonate factories dominated by cool-water biota was contemporaneous with a tectonically driven palaeogeographic change. To investigate this depositional shift and infer the relative impact of climate vs tectonics, a continental-scale sedimentological and geochemical study was conducted on twelve stratigraphic sections of DC strata from western Canada to southern Nevada (USA). The spatial–temporal distribution of microfacies records the turnover from [i] a Famennian lime mud-rich, shallow warm-water carbonate ramp with low sedimentation rates, mesotrophic conditions and tabular geometry to [ii] Tournaisian to Viséan lime mud-depleted and grainstone dominated cool-water carbonate ramp with anomalous high sedimentation rates, oligotrophic conditions and a pronounced slope. Positive excursions of *δ*^18^O_carb_ (+ 2‰ V-PDB) and *δ*^13^C_carb_ (+ 4‰ V-PDB) of Lower Mississippian carbonates likely correspond to the first cooling peak of the Carboniferous-Permian icehouse climate, following carbon withdrawal during black shale deposition during the late Famennian and early Tournaisian. However, late Tournaisian return of photozoan elements and their persistence throughout the Viséan suggests that warmer surface water existed, revealing a decoupling of the lower latitude ocean and the atmosphere. Shoaling of the thermocline was likely a result of cold-water upwelling along an open coast, as the Antler orogen no longer provided an oceanic obstruction to the west. This study shows that carbonate platforms are more susceptible to regional changes than global shifts.

**Supplementary Information:**

The online version contains supplementary material available at 10.1007/s10347-022-00653-4.

## Introduction

At the onset of the longest-lived (ca. 80 Ma), and likely most extensive and intense icehouse period of the Phanerozoic that started in the latest Devonian and spanned the Carboniferous Period (e.g., Isaacson et al. 2008; Montañez and Polsen 2013; Cheng et al. 2020), the lower latitude stratigraphic archive of western Laurentia (ancestral North America) recorded oceanic anoxia and major change in carbonate deposition (turnover). During the final stage of the Silurian-Devonian greenhouse period, western Laurentian seas hosted giant carbonate ramps with homogenous subtidal shallow-water facies, vast regional tidal-flat areas, and apparent layer-cake architecture such as the Palliser Formation in Alberta, Canada, and West-Range Formation in Utah and Nevada, USA (Read 1982; Kolata et al. 1991; Meijer-Drees 1994; Peterhänsel et al. 2008). These carbonate deposits were produced by phototrophic biota: stromatolites, calcispheres, tubular green algae and rare Labechiid stromatoporoids, and are typical of warm-water (> 20 °C) environments (Mamet 1975). After shutdown of carbonate factories in an episode of ocean anoxia and deposition of uppermost Devonian black shales (Caplan and Bustin 1999; Feist and Flajs 1988; Becker 1993; Kaiser et al. 2016), carbonate recovery occurred and coincided with the onset of the Carboniferous-Permian icehouse mode. Lower Mississippian ramps display cool-water (< 15 °C) carbonate characteristics with crinoid-rich deeper-water facies, a lack of regional tidal flats, and scattered isolated photozoan-dominated sediments that existed locally above a shallow thermocline (Read 1982; Brandley and Krause 1997; James 1997; Martindale and Boreen 1997). The few studies that have addressed the shift to colder-water carbonates in western Laurentia focussed only on Mississippian carbonates and concur in seeing a causal mechanism of carbonate turnover as being driven by regional upwelling of cold water, but these same studies did not fully address the role of climate (e.g., Martindale and Boreen 1997; Li et al. 2022). Differentiating climate change signals from regional environmental and oceanographic changes is a crucial step in understanding this late Palaeozoic Earth System shift, carbonate factory dynamics, and ultimately ocean response to global warming and cooling. Remarkable exposures of hundreds of metres thick Upper Devonian and Lower Carboniferous strata in cliff-forming outcrops extending from western Canada to the Great Basin of southern Nevada provide a bountiful record of depositional changes to investigate the main driver for carbonate turnover. Twelve outcrop sections along a 2100 km stretch from Alberta (Canada), Montana (USA) and Nevada (USA) were examined to document facies changes and investigate depositional dynamics of carbonates and associated architectural changes in carbonate ramp geometries along western Laurentia. Carbonate microfacies were used to establish new depositional models for the Upper Devonian and Lower Carboniferous strata. Coupling the spatial–temporal distribution of carbonate microfacies with whole-rock carbon and oxygen isotopic ratios (*δ*^13^C_carb_ and *δ*^18^O_carb_) in Mississippian carbonates as a proxy for palaeo-climate allows the testing of whether or not, climate cooling was the main driver or a contributing factor to carbonate turnover.

## Geological context

### Palaeogeographic and palaeotectonic setting of Western Laurentia

**Fig. 1:**
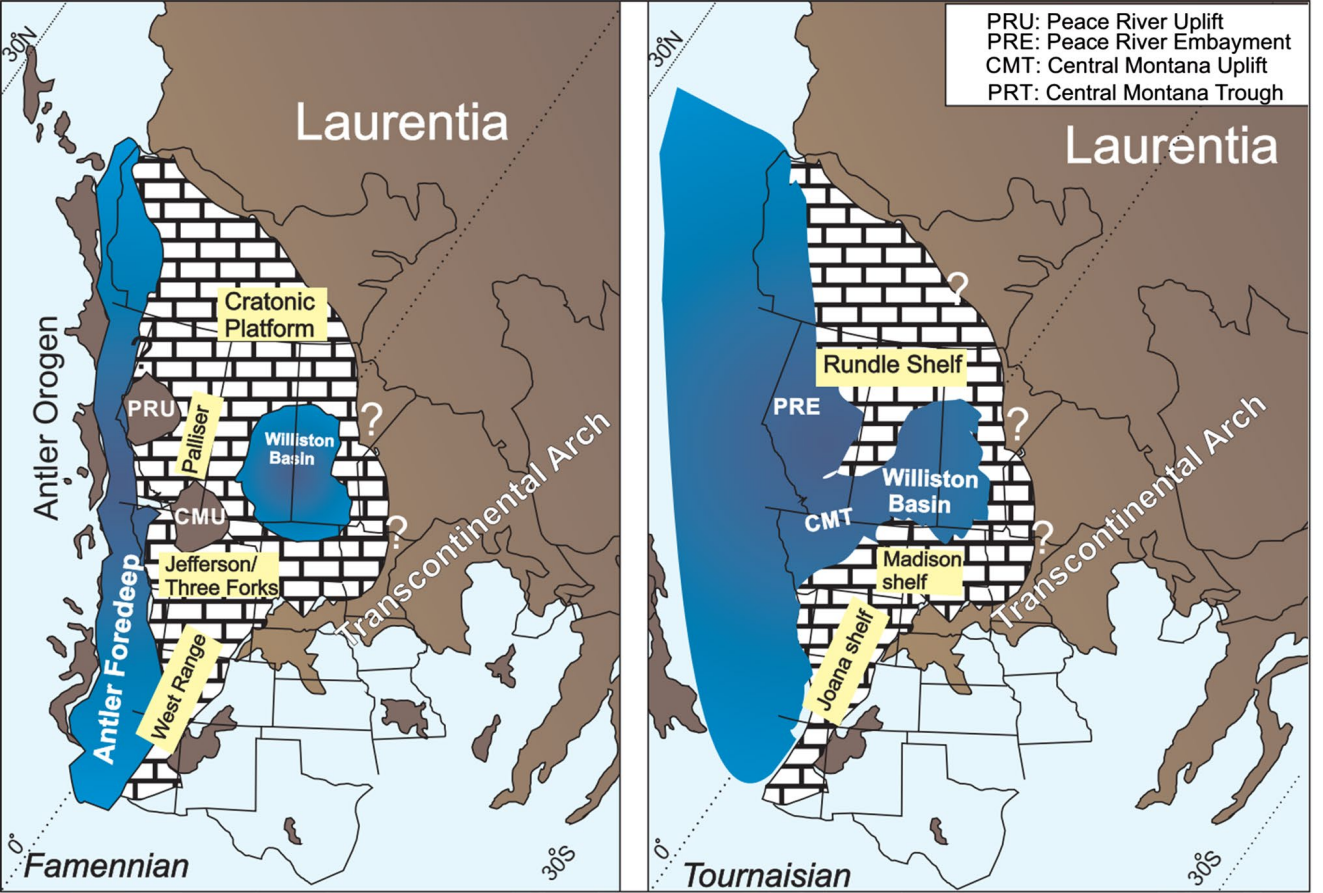
Palaeogeographic reconstruction and main structural elements of Western Laurentia during the Late Devonian (Famennian) and Early Carboniferous (Tournaisian). Note the pronounced deepening and structural inversion of uplifted areas (CMT and PRU) during the Tournaisian. Yellow boxes are names of stratigraphic units for that time interval

Palaeogeographic reconstructions of the Late Devonian indicate that the west coast of Laurentia occupied a near equatorial location (Blakey and Ranney 2017) (Fig. 1). Although the tectonic setting of western Laurentia is still unclear, growing evidence suggests that sediment deposition occurred in the incipient Antler Foreland Basin that extended from western Alberta to southern Nevada (Root 2001; Richards et al. 2002; Trexler et al. 2004; Hedhli et al. 2022). In western Canada, a shallow epicontinental sea covered the broad cratonic platform (Richards 1989) (Fig. 1). A discontinuous orogenic belt, known as the Cariboo Orogen, existed to the west and formed a northern extension of the Antler Orogen (Gordey et al. 1987). In northwestern United States, sediment deposition occurred in the Sappington Basin of the greater Central Montana Trough. In southwestern United States, uppermost Devonian to lowermost Carboniferous rocks of southern Nevada were deposited in the eastward-migrating Antler foreland basin (Giles and Dickinson 1995; Cook and Corboy 2004). During the early Tournaisian, western Laurentia was a site of pronounced regional subsidence caused by back-arc extension and block faulting (Richards 1989; Dorobek et al. 1993; Richards et al. 2002). Mississippian carbonate strata were deposited on a west-facing ramp that formed along western Laurentia (Banff, Rundle, Madison and Joana carbonate ramps) (Goebel 1991) (Fig. 1).

### Devonian to Carboniferous stratigraphy

**Fig. 2:**
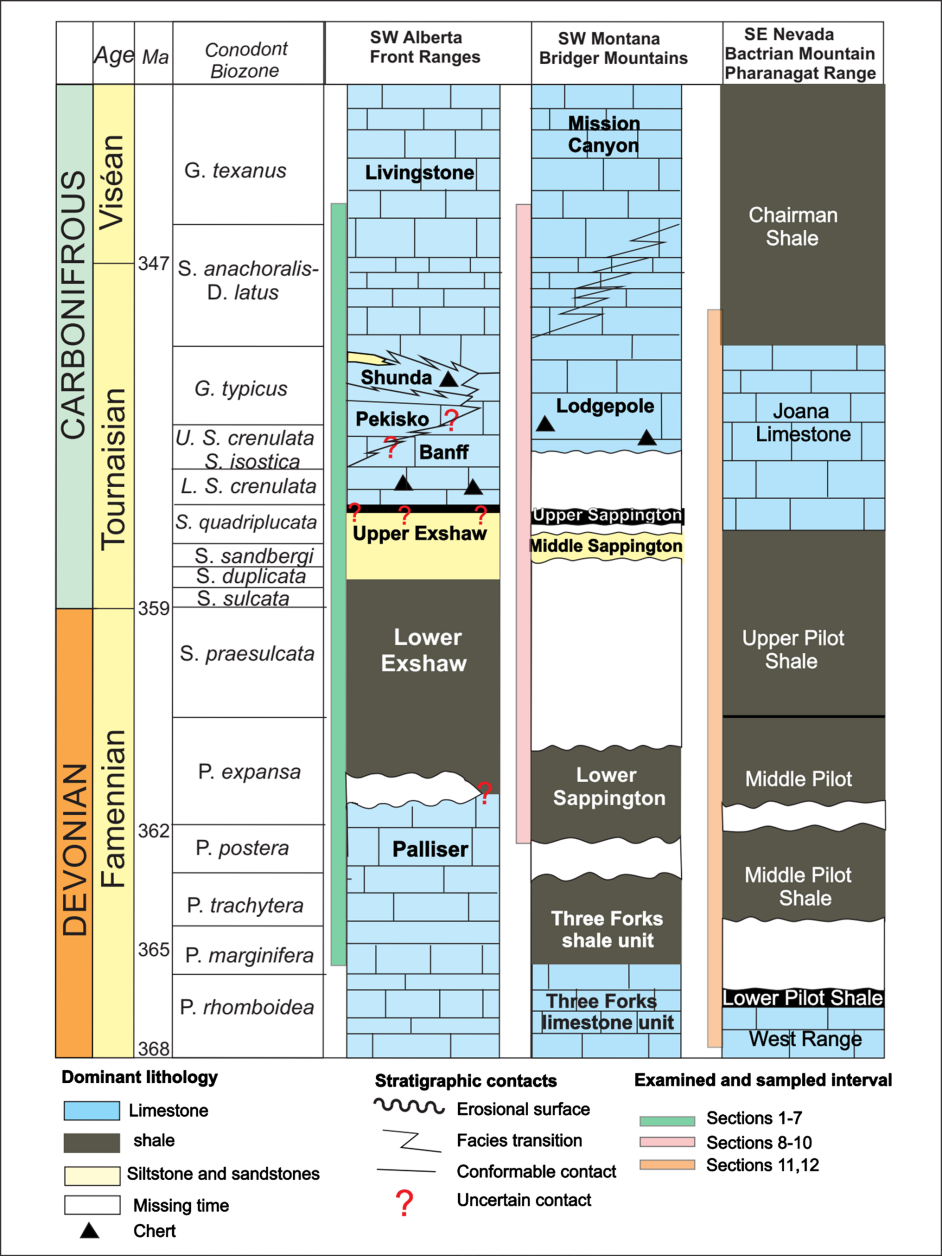
Biostratigraphic correlation of Upper Devonian to Lower Carboniferous litho-stratigraphic units in Alberta, Montana and Nevada modified from Sandberg (1979), Sandberg et al. (1980), Ziegler and Sandberg (1984), Richards et al. (1993) and Johnston et al. (2010). White areas represent hiatus due to non-deposition or erosion. Age in Ma is displayed in the 3rd column from left

Biostratigraphic ages and stratigraphic ranges are shown in Fig. 2 (Sandberg 1979; Sandberg et al. 1980; Ziegler and Sandberg 1984; Richards et al. 1993; Johnston et al. 2010). In Canada, Upper Devonian to Lower Mississippian strata are exposed in the southern and central Rocky Mountains. The thick limestone succession of the Palliser Formation is overlain by the upper Famennian to Tournaisian black shale and dolomitic siltstone of the Exshaw Formation. A thick succession of mainly limestone (i.e., Banff, Pekisko, Livingstone and Shunda formations) overlies the Exshaw Formation (Chatellier 1988; Richards et al. 1993). In southwestern Montana, the Upper Devonian to Lower Mississippian Sappington Formation is a mix of shale, siltstone, sandstone and limestone that unconformably overlies the Three Forks Formation (Fig. 2) (McMannis 1955). The Lodgepole Formation in Montana, consisting of thin- to medium-bedded carbonates with intercalated calcareous shale, is the equivalent to the Banff Formation in Alberta (Grover 1996). The Tournaisian to Viséan thickly bedded to massive fossiliferous and oolitic carbonates of the Mission Canyon Formation overlie the Lodgepole Formation (Peterson and MacCary 1987; Poole and Sandberg 1991). In Nevada, the Upper Devonian West Range Limestone is exposed in the mountain ranges of eastern Nevada where it conformably overlies the Guilmette Limestone. The West Range Limestone is time equivalent to the Three Forks Formation in Montana and to the Lower Member of the Palliser Formation in Alberta (Sandberg 1979; Johnston et al. 2010). Lower Mississippian carbonates of the Joana Limestone are extensively exposed in the mountain ranges of the Basin and Range Province in eastern Nevada and western Utah, where they overlie conformably the Famennian to Lower Tournaisian Pilot Shale. The Joana Limestone is time-equivalent to the Lodgepole Formation in Idaho and Montana.

### Study area

**Fig. 3:**
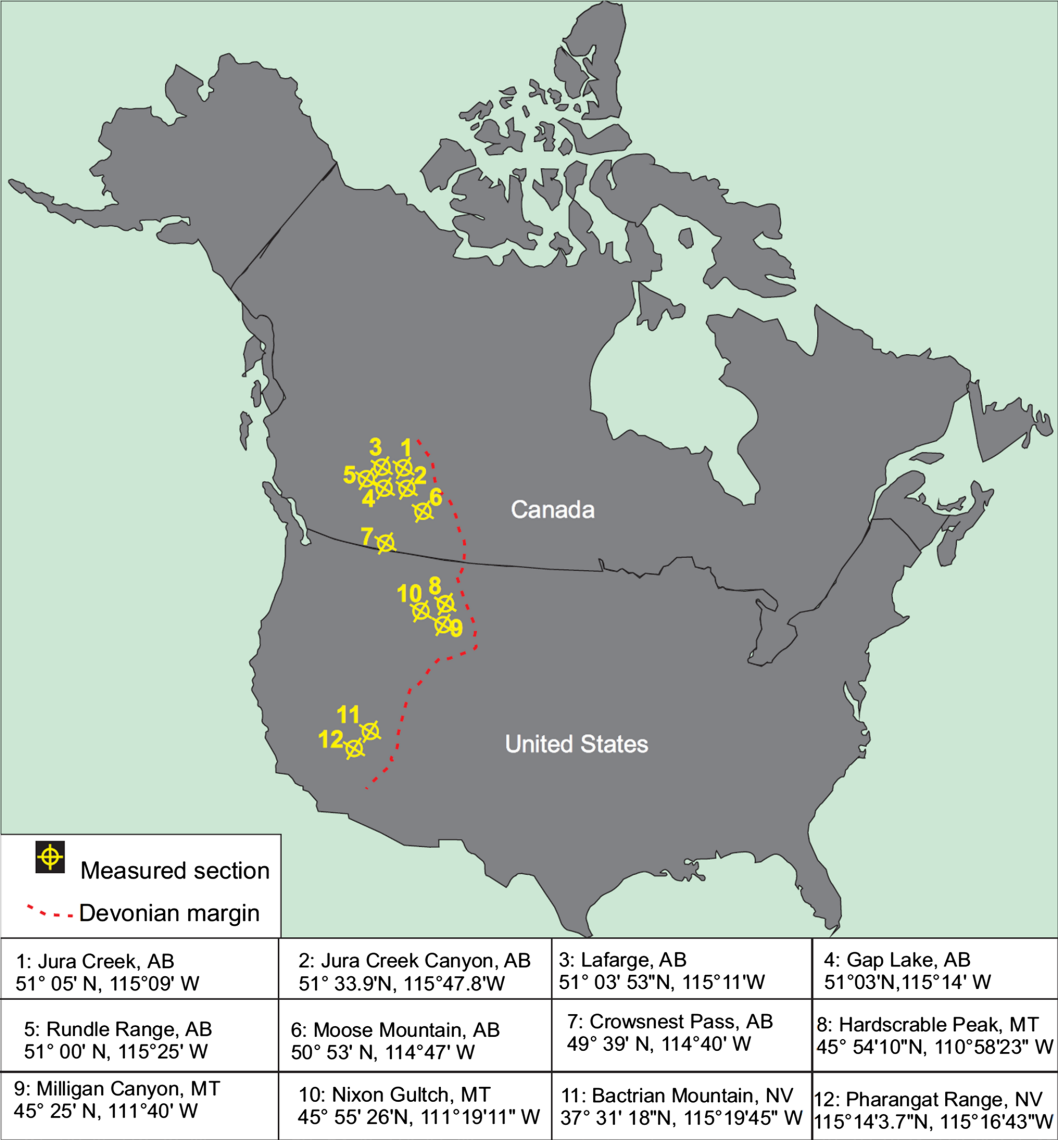
Map showing studied sections along a transect that represents the inferred Devonian to Carboniferous margin of western Laurentia

Twelve (12) stratigraphic sections were measured and sampled along a 2100 km segment extending from Alberta to Nevada. In Canada, studied outcrops (section 1 to 7) are in a series of thrust sheets along an east–west transect between the city of Calgary and town of Banff, and to the south in the Crowsnest Pass area near the Alberta/British Columbia border. In Montana, one outcrop section is located at the Bridger Range’s second highest point, known as the Hardscrabble Peak (section 8). Milligan Canyon and Nixon Gulch outcrops (sections 9 and 10) are located northeast of the town of Three Forks. In Nevada, the outcrop of the Bactrian Mountain is located near the town of Ash Springs (sections 11). The Pahranagat section is located west of the town of Alamo (section 12) (Fig. 3).

### Methods

For field sampling strategy, thin-section petrography and stable isotope sample preparation and analysis additional data are given in Online Resource 1 (Methods).

### Abbreviations

CCD: carbonate compensation depth; DC: Devonian to Carboniferous; FWWB: fair weather wave base; MF: microfacies; SWB: storm wave base; OMZ: oxygen minimum zone; PZ: photic zone; TC: thermocline.

## Microfacies

Microfacies analysis refers to discerning the sedimentological and palaeontological data which can be described and classified from thin-sections and rock samples (Flügel 2004). Eleven (11) carbonate microfacies (MF1 to MF11) were recognized in the uppermost Devonian and Lower Mississippian carbonate rocks and were classified using the Dunham (1962) classification. A summary of the relationships between these microfacies is provided in Table.1. [Examples of detailed stratigraphic columns and facies analysis are given in Online Resource 2].

**Table 1:** Upper Devonian and Lower Mississippian microfacies of Western Laurentia and depositional environments

Microfacies	Petrographic description	Gain assemblage	Field observations	Depositional environment
MF1-Silty lime mudstone	Fabric: lime mudstone Common to abundant quartz, organic matter and trace fossils. Rare chert	Rare sponge spicule, echinoderm, bryozoan and brachiopod fragments	Occurs in thin to medium bedded dark grey limestone that weathers dark yellow	Cool, deep-water environment with low energy and low to normal oxygen levels. Below PZ, TC and SWB. Basin
MF2- Spiculitic radiolarian lime mudstone	Fabric: lime mudstone with localized wackestone. Common to abundant chert and bioturbation. Rare to common dolomite	Abundant sponge spicule. Common radiolarians. Rare ostracods, echinoderm, bryozoan and brachiopod fragments	Occurs in dark coloured thinly-bedded argillaceous limestone	Cool and deep water with low energy and relatively high sedimentation rates. Below PZ and thermocline. Outer-ramp: lower slope
MF3- Bryozoan crinoidal wackestone	Fabric: wackestone with subordinate packstone. Grains fragmented but not abraded	Abundant crinoid and bryozoan. Common brachiopod and ostracod fragments. Rare trilobite, sponge spicule and red algae	Occurs in dark coloured thinly-to medium bedded resistant limestone	Cool and deep water setting with low energy and moderate sedimentation rates. Below the thermocline and below the SWB. Outer ramp: middle to upper slope
MF4- Crinoidal packstone-grainstone	Fabric: packstone and grainstone with minor wackestone. Grains are fragmented and abraded	Crinoids are dominant. Common bryozoan and brachiopod fragments. Rare to common foraminifer Earlandia sp., sponge spicule and red algae	Occurs in light grey, medium to thick bedded, resistant limestone	Cool and relatively moderately deep water setting with intermittent but relatively frequent high energy. Below the thermocline and above the SWB. Mid-ramp
MF5- Crinoidal algal grainstone	Fabric: grainstone with minor packstone. Grains are micritized and abraded. Rare Chert and dolomite	Abundant crinoids and red algae. Common bryozoan and brachiopod fragments. Rare to common: foraminifer Earlandia sp., and reworked green algae	Occurs in the. found in medium to thick bedded, cliff forming light grey limestone	Cool and relatively shallow water setting with relatively frequent and high energy. Below the thermocline, above the SWB but below the FWWB in open marine realm. Proximal mid-ramp
MF6-Oolitic wackestone	Fabric: wackestone with minor packstone. Grains are micritized and abraded. Common intraclasts	Common ooids, bryozoan and brachiopod and bivalve fragments. Rare to common trilobites, ostracods and green algae	Occurs in medium to thick bedded grey limestone	Texturally inverted: grains developed in warm and relatively shallow water setting but resedimented in relatively low energy environment near the thermocline, below the FWWB. Inner to mid-ramp transition /fore shoal
MF7-Oolitic bioclastic grainstone	Fabric: grainstone with minor packstone. Grains are micritized and abraded. Common Fibrous marine cement	Ooids are dominant: Common to abundant: calcareous green algae, crinoid, echinoid, bryozoan, brachiopod and bivalve fragments. Rare to common: Foraminifera, peloids, and trilobite and ostracod fragments	Occurs in medium to thickly bedded light grey limestone	Warm and very shallow water setting. High frequency and intensity water energy. Above the thermocline and the FWWB. Inner-ramp/shoal
MF8- Peloidal packstone	Fabric: packstone with minor grainstone	Peloids are dominant. Common oncoids with Girvanella sp. Rare to common bryozoan gastropod, trilobite, ostracod, calcareous green algae, crinoid, echinoid, brachiopod and bivalve fragments	Occurs in medium to thickly bedded light grey limestone	Warm and very shallow water setting. Moderate energy. Above the thermocline and FWWB. Semi protected environment. Inner ramp: back shoal/lagoon
MF9- Algal wackestone	Fabric: wackestone with minor packstone	Abundant: calcareous green algae: calcisphere, Proninella sp., Issinella sp., Pseudokamaena sp. Common ostracods, crinoids, and ammonoids. Rare brachiopod and bivalve fragments	Occurs in medium bedded dark grey limestone	Warm and shallow water setting with low energy. Above the thermocline, at or below the FWWB. Protected inner-ramp: shallow subtidal zone
MF10-Charophyte algal wackestone	Fabric: wackestone	Abundant charophytes, calcareous green algae: calcispheres and other tubular algae. Common intact gastropods. Rare ostracod and bivalve fragments	Occurs in dark grey coloured, medium bedded limestone	Warm and shallow water setting with moderate energy. Above the thermocline and at the FWWB. Protected inner ramp: shallow subtidal to intertidal
MF11-Peloidal-fenestral mudstone-boundstone	Fabric: fenestral fabric in a mudstone matrix with localized boundstone. Stromatolitic and thrombolytic textures are visible	Abundant peloids calcisphere, tubular green algae common oncoids, calcispheres and ostracods	Occurs in dark grey coloured, medium bedded limestone	Warm and very shallow water setting intermittently exposed. Low energy but above the FWWB. Restricted to Protected-Inner-ramp: tidal flats, intertidal to supratidal

### MF1-Silty lime mudstone

**Fig. 4:**
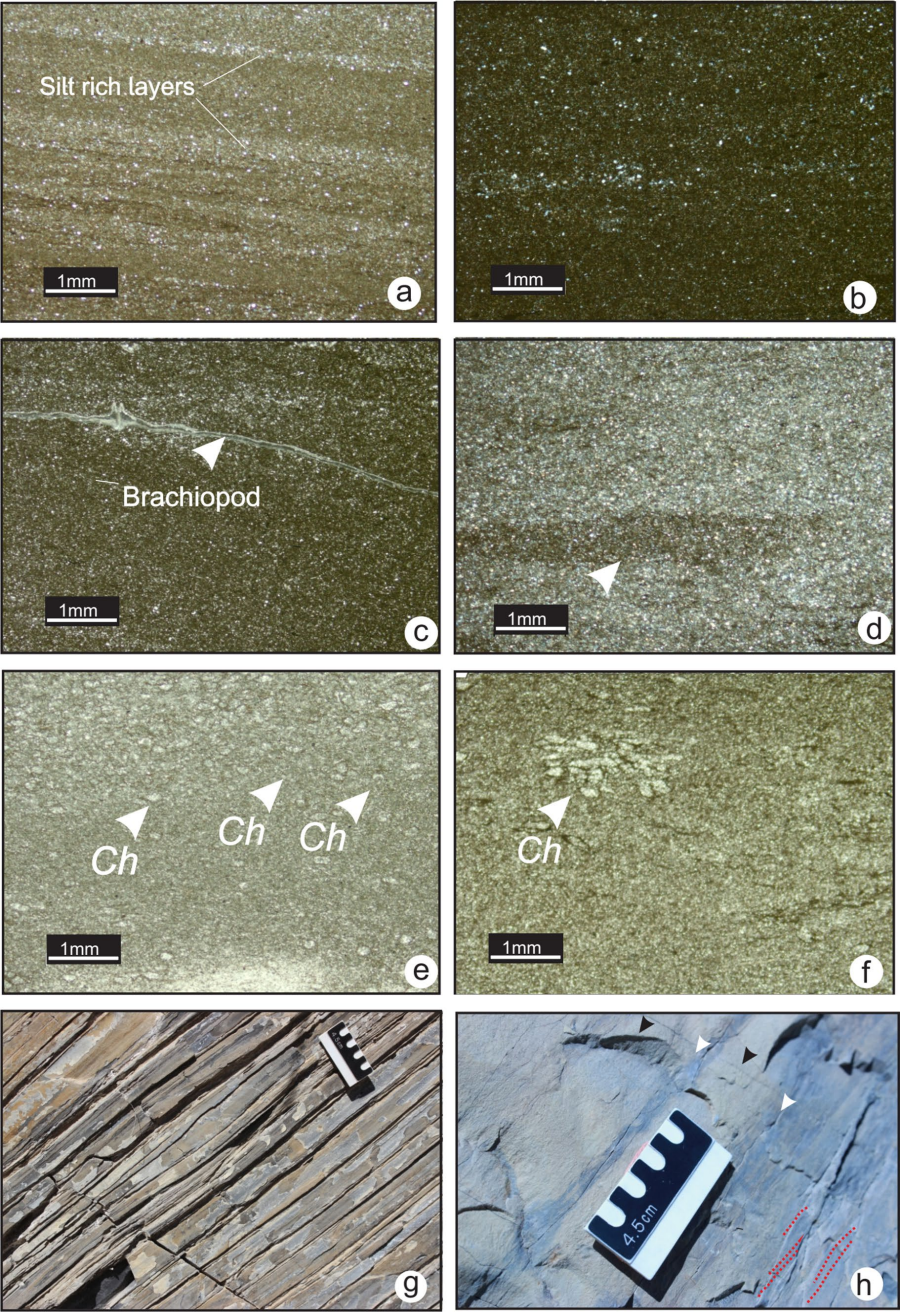
Photomicrographs of MF1 from lower Banff Formation. **a** Laminae and quartz rich layers (TS 3–25, section 3). **b** Sparse silt-sized quartz grains in a lime mudstone matrix (TS 3–28, section 3). **c** Brachiopod shell (TS 3–25, section 3). **d** Arrow points to bioturbation (*Helminthopsis* isp). **e** and **f** *Chondrites* isp*. (Ch)* (TS 3–55 and TS 3–56, section 3). **g** Rhythmic thin beds of the lower Banff Formation at section 3. **h** White arrows point to mud-rich layers, black arrow points to silt-rich layers (MF1), Gently dipping cross lamination outlined in red

MF1 is dominated by lime mud and contains very fine-sand to silt-sized quartz grains both dispersed as well as forming discrete laminae (Fig. 4a). Wavy to planar laminae are present (Fig. 4b) with scarce sponge spicules, small fragments of crinoids and brachiopods (Fig. 4c). Bioturbation is common (Fig. 4d), but trace fossils are low in diversity and are dominated by *Chondrites* isp. (Fig. 4e and f). The relatively dark coloured matrix reflects the presence of disseminated organic matter. MF1 is present in the lower Banff Formation of southwestern Alberta and in the Lodgepole Formation of southern Montana. In the field, this microfacies occurs as a thin to medium rhythmically bedded dark-grey limestone that weathers dark yellow (Fig. 4g). These thin beds display gently dipping cross lamination (Fig. 4h).

The lime mud dominance in MF1 is interpreted to indicate a low-energy, deeper-water setting below the SWB and the PZ. Low diversity and abundance of fossils with rare sponge spicules, crinoids and bryozoans indicate basin floor to lower slope environments with low hydrodynamic energy. Dominance of horizontal trace fossils such as *Chondrites* isp*.* are indicative of dysaerobic conditions. Rhythmicity of beds is created by alternation of silt-rich and lime-mud rich layers. This interbedding could reflect the interplay between carbonate accumulation and hemipelagic deposition of fine sediments as a result of a change in carbonate production or fluctuation in allochthonous sediment flux. In southwestern Alberta and Montana, occurrences of subtle slump features at outcrop suggest that these beds correspond to distal turbidites on a gently-dipping slope (Chatellier 1988; Richards et al. 1993).

### MF2-Spiculitic-radiolarian lime mudstone-wackestone

**Fig. 5:**
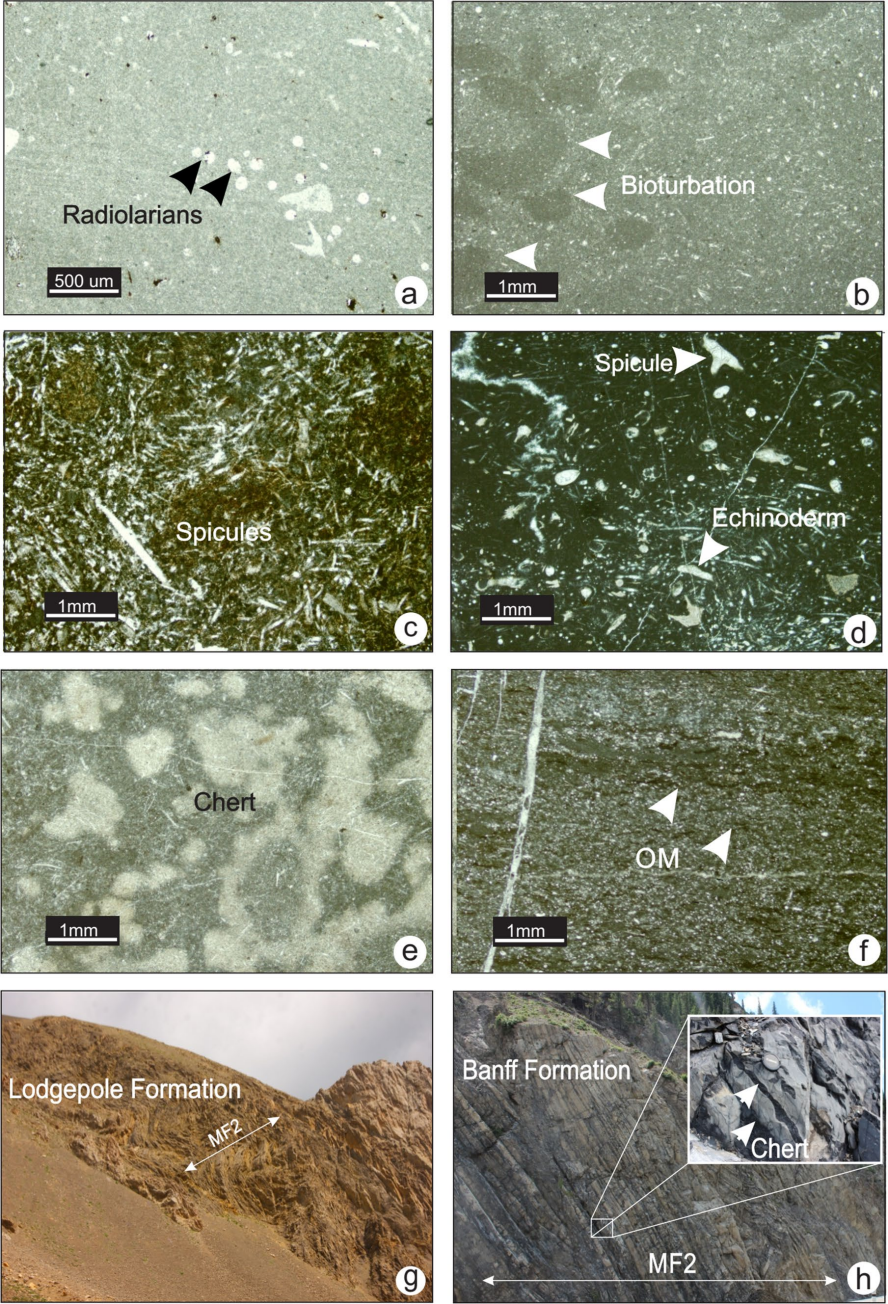
Photomicrographs of MF2 from lower Lodgepole, Banff and Joana formations. **a** Calcitized radiolarians (small white spheres) in Lodgepole Formation (TS LP-12,section 8). **b** Bioturbation in the Lodgepole Formation (TS LP-12, section 10). **c** Spiculitic wackestone in the upper Joana Limestone (TS PH-78, section 12). **d** Spiculitic wackestone with echinoderm and bryozoan fragments in the upper Joana Limestone (TS BM-140 and TS BM-153, section 11). **e** Cherty intervals in the Banff Formation (TS 6–102, section 7). **f** Organic matter (OM) in the Banff Formation (TS 6–146, section 7). **g and h** Photographs of outcrops showing the extent of MF2. Thinly-bedded limestone with abundant chert nodules in the lower Lodgepole (**h**, section 8) and Banff formations (**g**, section 7)

MF2 consists of a medium grey micritic matrix with variable amounts of sponge spicules and common to abundant radiolarians (Fig. 5a). Sponge spicules are sparse or concentrated in laminae (Fig. 5c and d), with rare echinoderm, bryozoan and ostracod fragments in a wackestone fabric (Fig. 5d). Laminae and bioturbation are common (Fig. 5b). Diagenetic chert is common to abundant in certain intervals (Fig. 5e). Organic matter is disseminated and amorphous or occurs along fractures and dissolution seams (Fig. 5f). MF2 is generally observed in dark coloured thinly-bedded argillaceous limestone units within the lowermost Mississippian strata. At outcrop, chert is commonly present as multi-decimetre cylindrical nodules or thinly interbedded with the limestone (Fig. 5h). This microfacies is mainly present in the Banff, Lodgepole and Joana formations (Fig. 5g).

The association of radiolarians with spicules is indicative of deep bathymetry. Accumulations of sponge spicules are due to either *in-situ* deposition after the disintegration of soft-body parts or through transport of spicules to the deep basin by currents, especially when parallel orientation of spicules is observed (Jach 2002). The sponge spicules and radiolarians are probably autochthonous or para-autochthonous; however, other fossil fragments are likely allochthonous transported by bottom currents. MF2 was likely deposited in a cold deep-water low-energy environment below the SWB and the PZ (above the CCD).

### MF3-Bryozoan crinoidal wackestone-packstone

**Fig. 6:**
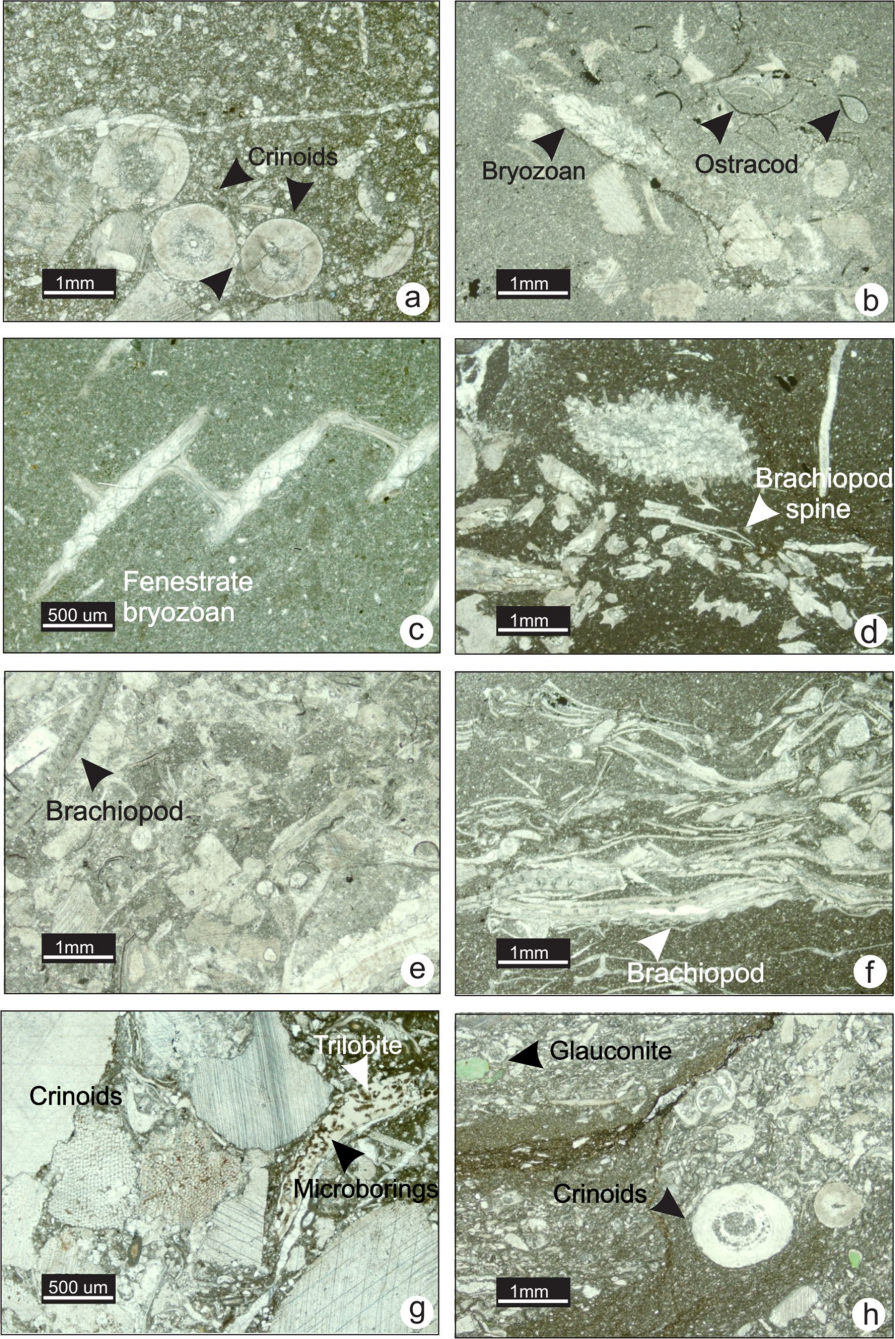
Photomicrographs of MF3 (crinoidal wackestone) from Lodgepole and Banff formations. **a** Crinoidal fragments (TS LP-26, section 8). **b** Ostracod, bryozoan and echinoderm fragments in a lime mudstone matrix (TS LP-26, section 8). **c** Fenestrate bryozoan fragment (TS NG-30, section 9). **d** Bryozoan fragment in wackestone fabric (TS 3–94, section 3). **e**. Well-preserved pseudo-punctate brachiopod shell (TS 3–89, section 3). **f** Crinoid and brachiopod debris in storm deposit (TS 3–94, section 3). **g** and **h** Unusual occurrences of packstone with trilobite, crinoid and ostracod fragments in the lower Banff Formation (TS 6Lg-4, section 7)

MF3 is characterized by low fossil diversity in a dominantly wackestone fabric with minor packstone. Fenestrate bryozoans and crinoids are the main fossils found (Fig. 6a and b); brachiopods are rare to common (Fig. 6c and e), ostracods, red algae and sponge spicules are present but rare (Fig. 6d). In the field, this microfacies occurs in moderately resistant, medium bedded, medium-to-dark grey limestone. The bryozoan crinoidal wackestone is commonly found alternating with spiculitic lime mudstone (MF2) and crinoidal packstone-grainstone (MF4) (Fig. 6b and g). Bioclasts lack signs of abrasion, and some fenestrate bryozoans are partially intact (Fig. 6c). Locally in southern Alberta, and southern Montana, MF3 displays a packstone fabric with abundant crinoid ossicles, glauconite, trilobite fragments and ostracods with microborings on their surfaces (Fig. 6f, g and h).

Microboring of the grains suggests a considerable residence time of the grains on the seafloor and low sedimentation rates. The heterotrophic fossil assemblage and texture of MF3 is indicative of a cool, low-energy environment below the thermocline. The presence of lime mudstone and sponge spicules suggests a close spatial relationship between MF3 and MF2 (radiolarian-spiculitic lime mudstone to wackestone). During periods with low sedimentation rate, bryozoans and rare brachiopods populated the substrate**.** Intact bryozoans and crinoids may have been fragmented in situ*,* indicating they were neither heavily reworked nor transported for considerable distances. Where the packstone fabric occurs, alignment and poor sorting of fossil fragments suggest deposition below but near the SWB on the outer ramp. This deeper-water packstone might have been deposited above the SWB but further transported by gravity into a lower energy environment below the SWB and accumulated during periods of sediment starvation.

### MF4-Crinoidal packstone-grainstone

**Fig.7:**
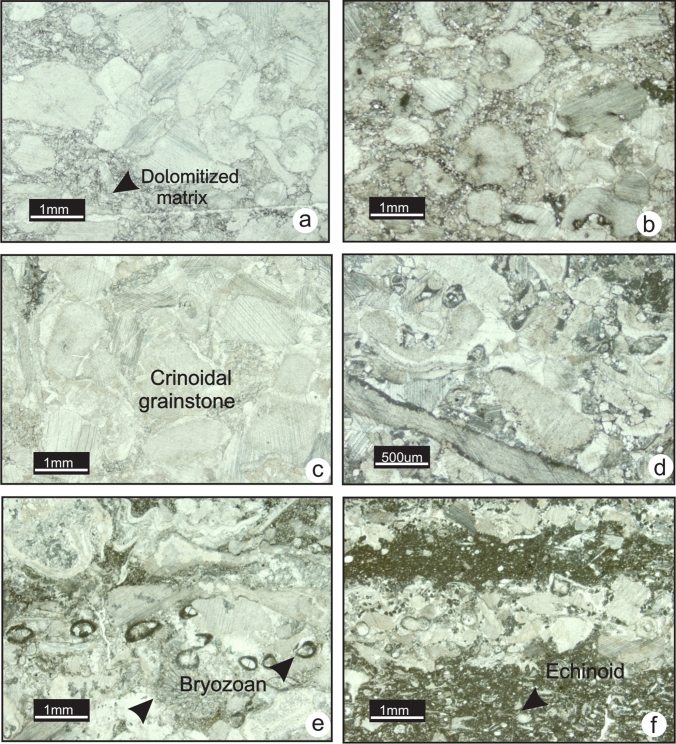
Photomicrographs of grainstone fabric in MF4. **a**. Crinoidal grainstone in the Pekisko Formation (TS 8–74, section 6). **b** Crinoidal grainstone with common fenestrate bryozoans in the Joana Limestone (TS Ph-75, section 12). **c.** Crinoidal grainstone with rare foraminifer *Earlandia* in the Pekisko Formation (TS 3–108, section 3). **d** Bryozoan and crinoid fragments in the Pekisko Formation (TS 3–127, section 3). **e** and **f** Different stages of dolomitization of crinoidal grainstone with xenotopic and idiotopic dolomite crystals (TS 5–92 and TS 5–93, section 4)

**Fig. 8:**
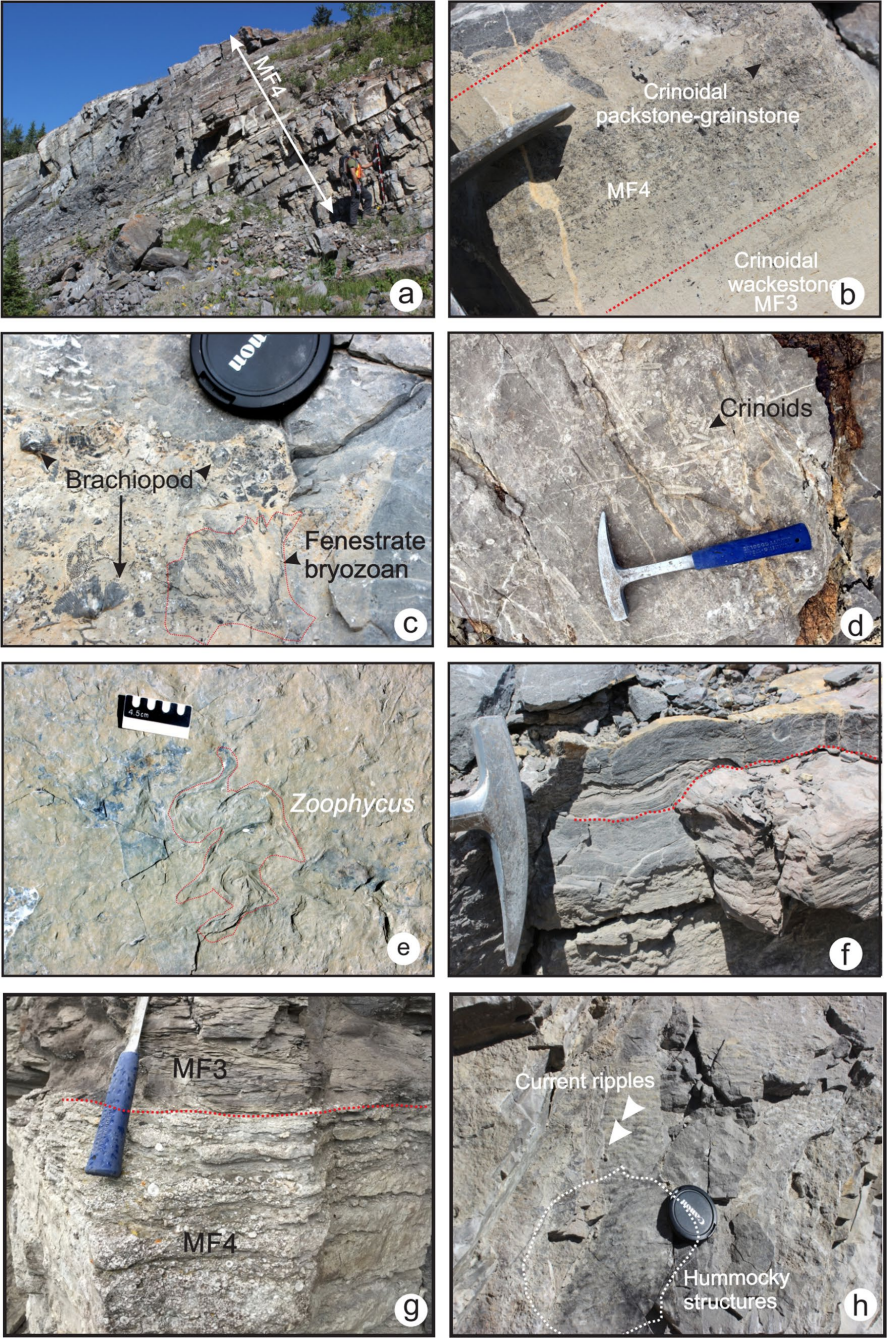
**a** and **g** MF4 in the middle Banff Formation (section 4). **b** Tempestites consisting of alternations of crinoidal wackestone and packstone in the middle Banff Formation (section 3). **c** Intact bryozoan and brachiopod in the Banff Formation (section 3). **d** Crinoidal stems in the Joana Limestone (section 11). **e** *Rhizocorallium*. isp. in the middle Banff Formation at section 3. **f** Erosional surfaces in the middle Banff Formation (section 4). **g** Erosional contact between MF3 and MF4 at section 6. **h** Hummocky bedforms and current ripples in the Banff Formation (section 3)

This microfacies displays grain-supported fabrics ranging from packstone to grainstone (Fig. 7a and b). Crinoid fragments make up 90–100% of the grains. Common bryozoan fragments, rare echinoids, brachiopods and foraminifera *Earlandia *sp. are present (Fig. 7c and d). Some grains are heavily fragmented, broken and abraded, whereas others are intact and not abraded. However, compaction and grain-to-grain dissolution might mask the roundness of the grains in the grainstone. Diagenetic processes affecting this microfacies include compaction, chertification and dolomitization. The texture of dolomite varies from anhedral crystals to subhedral crystals (idiotopic) (Fig. 7e and f). The matrix of this microfacies is particularly dolomitized in the Banff Formation. In the field, this microfacies occurs as medium to thick-bedded, light-grey coarse-grained limestone alternating with recessive argillaceous limestone beds. MF4 is commonly interbedded with crinoidal wackestone and packstone (MF3). Large and small-scale hummocky cross-stratification and erosional surfaces are common in these units (Fig. 8f and h). Resedimented solitary rugose corals, fenestrate bryozoans and spiriferid brachiopods are common to abundant at outcrop (Fig. 8c, d and g). Fossiliferous beds scour underlying beds. Trace fossils are scarce; only rare occurrences of *Rhizocorallium* isp. are observed at outcrop (Rodriguez and Gutschick 1970) (Fig. 8e). MF4 is found in all the studied sections where it forms prominent cliffs of carbonate. However, it is thicker in Alberta and Montana where it reaches up to 40 m.

The grain-supported aspect of this microfacies indicates that it formed by mass accumulations of crinoids in a high-energy environment. The low volume of lime mud suggests high intensity winnowing and sporadic reworking during deposition, although the presence of mud-rich intervals in the field suggests periods of quiescence. Except for the increase in the grain proportion relative to the matrix, this microfacies is similar to MF3 (crinoidal-bryozoan wackestone-packstone), and MF4 may have been a proximal lateral continuation of MF3. The poor sorting of clasts and abrasion of fossils support the interpretation of variable transport distances. Intact bryozoans and brachiopods seen in outcrop might have been deposited in situ with minor reworking, whereas crinoids might have been transported from a shallower-water environment or from nearby crinoid banks. Overall, MF4 reflects deposition above the SWB. Erosional contacts between beds and hummocky cross-stratification observed in the field are characteristic of storm deposits. The thickness of the grainstone beds suggests high frequency and high intensity of swells.

### MF5-Algal crinoidal grainstone

**Fig. 9:**
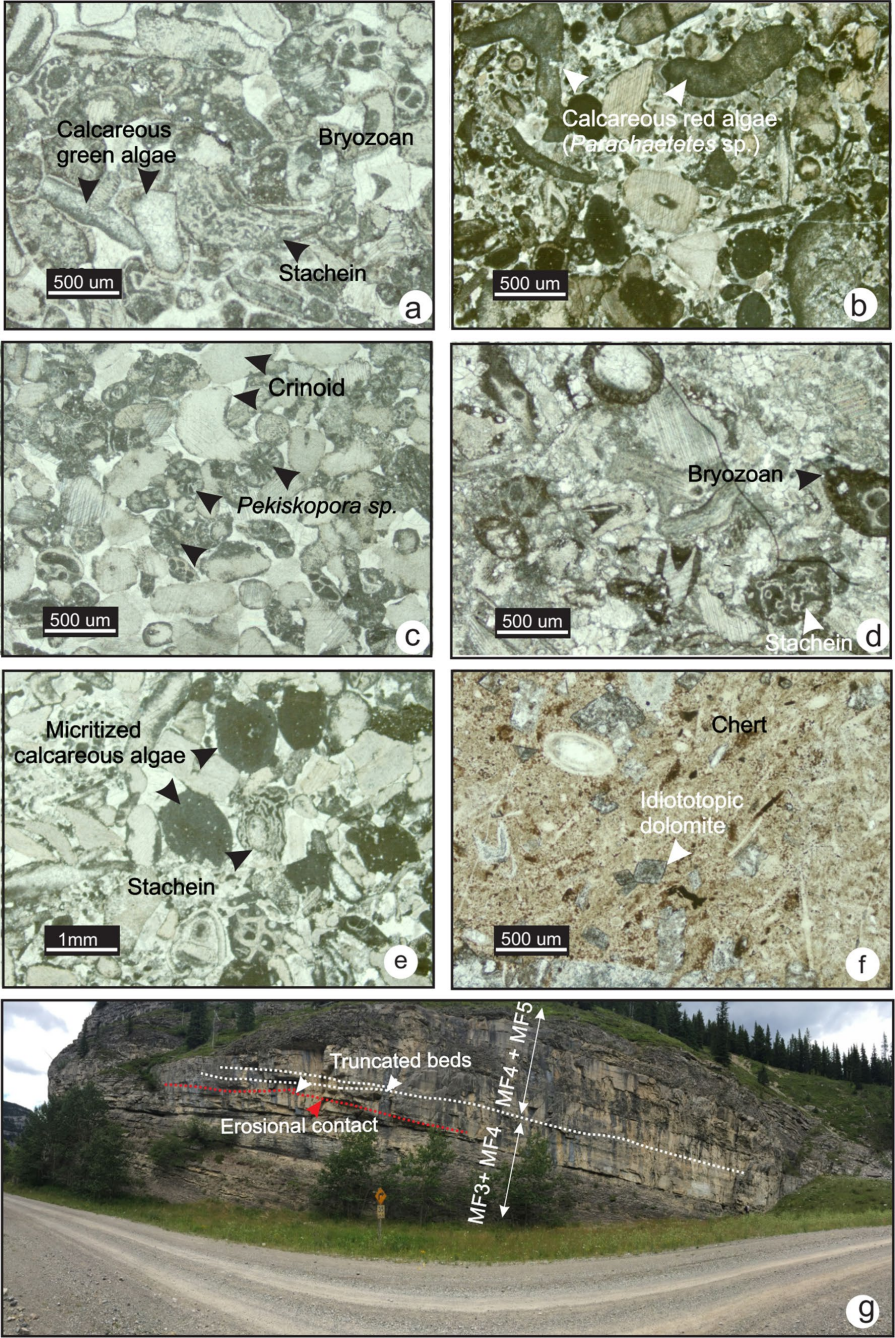
Photomicrographs of MF5 in the Pekisko Formation. **a** Algal-crinoidal grainstone with red and green calcareous algal fragments (TS 3–117, section 3). **b** Fragments of *Parachaetetes* sp. (TS 8–75, section 6). **c** *Pekiskopora* sp. in a packstone (TS 3–104, section 3). **d** Stacheinaceae and bryozoan (TS 8–93, section 6). **e** Micritized grains (TS 8–93, section 6). **f** Silicified idiotopic dolomite (TS 3–124, section 3). **g** Thickly-bedded hummocky cross-stratified limestone with truncated beds in the Pekisko Formation at Moose Mountain (section 6). Notice the erosional Banff/Pekisko contact (red line)

MF5 is a grainstone, dominated by crinoid ossicles, and red and green algae (Fig. 9a). Packstone fabrics are rare. Crinoid ossicles are abraded and affected by pervasive micritization. Algal fragments are abundant and consist mainly of stacheinacean algae and other algal groups (Mamet 1975) including common *Pekiskopora *sp.,* Parachaetetes *sp. and rare *Tubisalebra *sp., (Fig. 9b and c). Fragments of fenestrate bryozoans, brachiopods and solitary rugose corals are common (Fig. 9d and e). Fragments of calcareous green algae and unidentified micritized grains are also common. Silicified coarse crystalline euhedral dolomite occurs in chert-rich intervals (Fig. 9f). In the field, this microfacies occurs in thick-bedded light grey, coarse-grained limestone. Large and small-scale hummocky cross-stratification and erosional surfaces and truncations are common in the units where MF5 occurs (Fig. 9g). MF5 is usually found interbedded with the crinoidal grainstone of MF4. This microfacies is found in southwestern Alberta but not observed in Montana or Nevada. The grainstone fabric of this microfacies suggests deposition in a high-energy environment. The biota in this microfacies is heterotrophic and reflects a heterozoan assemblage deposited in cool water below the thermocline. However, the presence of minor photozoan elements such as fragments of resedimented green algae, micritized grains and endolithic micritized envelopes around the grains indicates that MF5 was affected by the thermocline fluctuation and sporadic influx of warm waters and formed a heterozoan-extended assemblage. Alternatively, the photozoan elements may have been transported offshore from a shallower, more proximal environment above the thermocline.

### MF6-Oolitic bioclastic wackestone-packstone

**Fig. 10:**
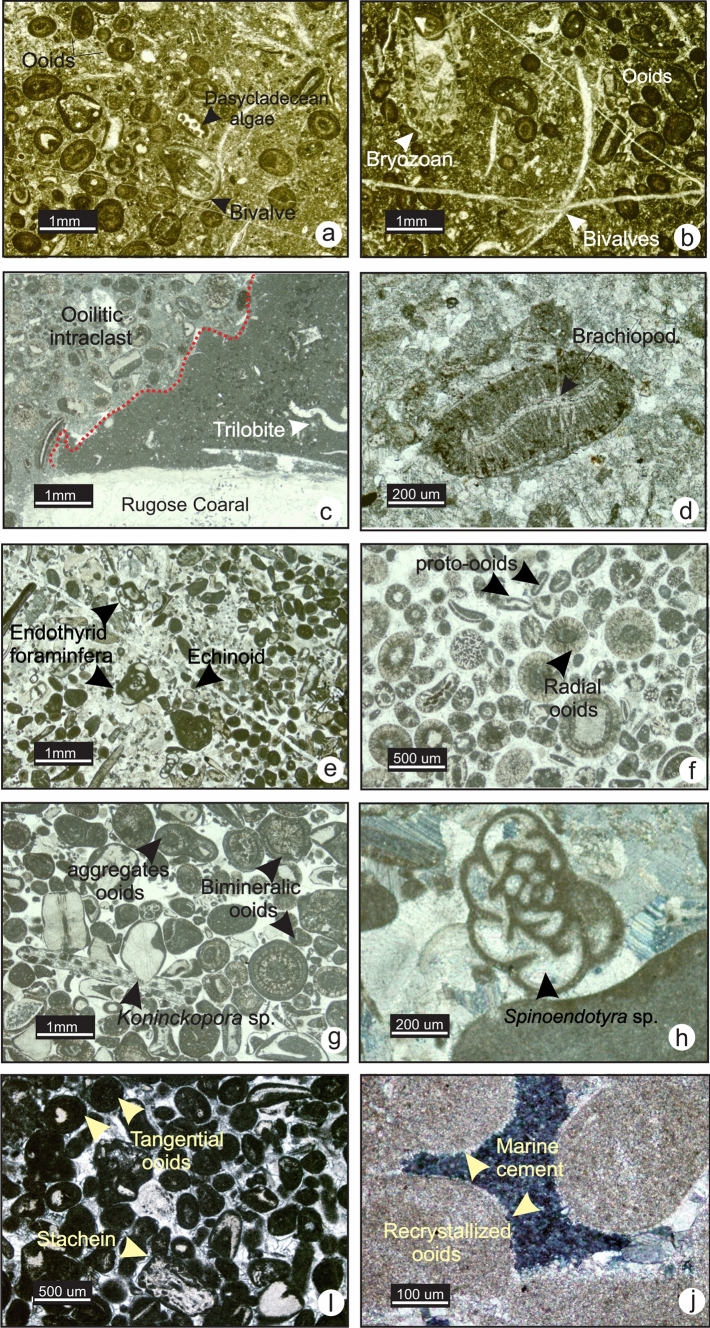
Photomicrographs of MF6 and MF7. **a** and **b** Oolitic packstone wackestone with green calcareous algae, bivalve and bryozoan fragments in the Palliser Formation (TS 1–5, section 1). **c** Oolitic intraclast in a bioclastic wackestone with trilobite(?) from the Mission Canyon Formation (TS MC-10, section 8). **d** Ooid with brachiopod fragment as a nucleus in the lower Banff Formation (TS 8–15, section 6). **e** Oolitic bioclastic grainstone (TS 8–96, section 6). **f.** Radial and bimineralic ooids in the Mission Canyon Formation (TS. MC-8, section 8). **g** Lump, bimineralic ooids and alga *Koninckopora* sp. fragment in the Mission Canyon Formation (TS MC-12, section 8). **h** Foraminifera *Spinoendothyra* sp*.* (TS 8–96, section 6). **i** Tangential ooids in the Joana Limestone (TS BM-80, section 11). **j** Fibrous marine cement rimming radial ooids in the Joana Limestone (TS BM-80, section 11)

MF6 is characterized by wackestone to packstone fabrics. The matrix is micritic and partially recrystallized. Common bioclasts are bryozoan, echinoderm, brachiopod and bivalve fragments, and calcareous green algae. Dasycladacean and tubular green algal fragments are commonly surrounded by a micritic envelope (Fig. 10a). Non-skeletal grains in this microfacies are abundant ooids ranging from 0.25 to 1.5 mm in diameter (Fig. 10a to d), and common intraclasts (Fig. 10c). In the field this microfacies occurs in light grey coloured thick-bedded limestone with macrofossils including ammonoids, stromatoporoids, bryozoans and crinoids. MF6 occurs in the upper Famennian and in the Viséan carbonates.

The photozoan assemblage of MF6 reflects development in warm water above the TC. However, this microfacies is marked by textural inversion. Ooids usually form in high-energy environments above the FWWB. Yet, the presence of substantial amounts of lime mud in MF6 suggests a moderate to low-energy environment below the FWWB. In modern environments, low-energy ooids have been found in lagoons, coastal lakes and protected shallow environments with different salinity (Sass et al. 1972; Hesse 1973). For instance, low-energy oolitic sands with carbonate mud occur in lagoons along the Sinai beaches of the Gulf of Suez (Sass et al. 1972) and are interpreted to be wind-blown ooids. Ooids in deep-water settings are commonly allochthonous shallow-water ooids transported in debris flows or turbidity currents. Deep-water ooids can also be “pelagic ooids” found on the top of drowned platforms and seamounts (Hesse 1973). Ooids found in MF6 are fine to coarse-sand sized and occur in intraclasts that reflect resedimented pre-existing lithified ‘allochthonous shallow-water ooids’ from a nearby shoal environment or might have accumulated on a drowned platform below the FWWB. The diversity of fossils in MF6 suggests an overlap between the heterozoan and photozoan assemblages with photozoan dominance. Such an environment exists in a fore-shoal or back-shoal environment and can extend to the mid ramp.

### MF7-Oolitic bioclastic grainstone

**Fig. 11:**
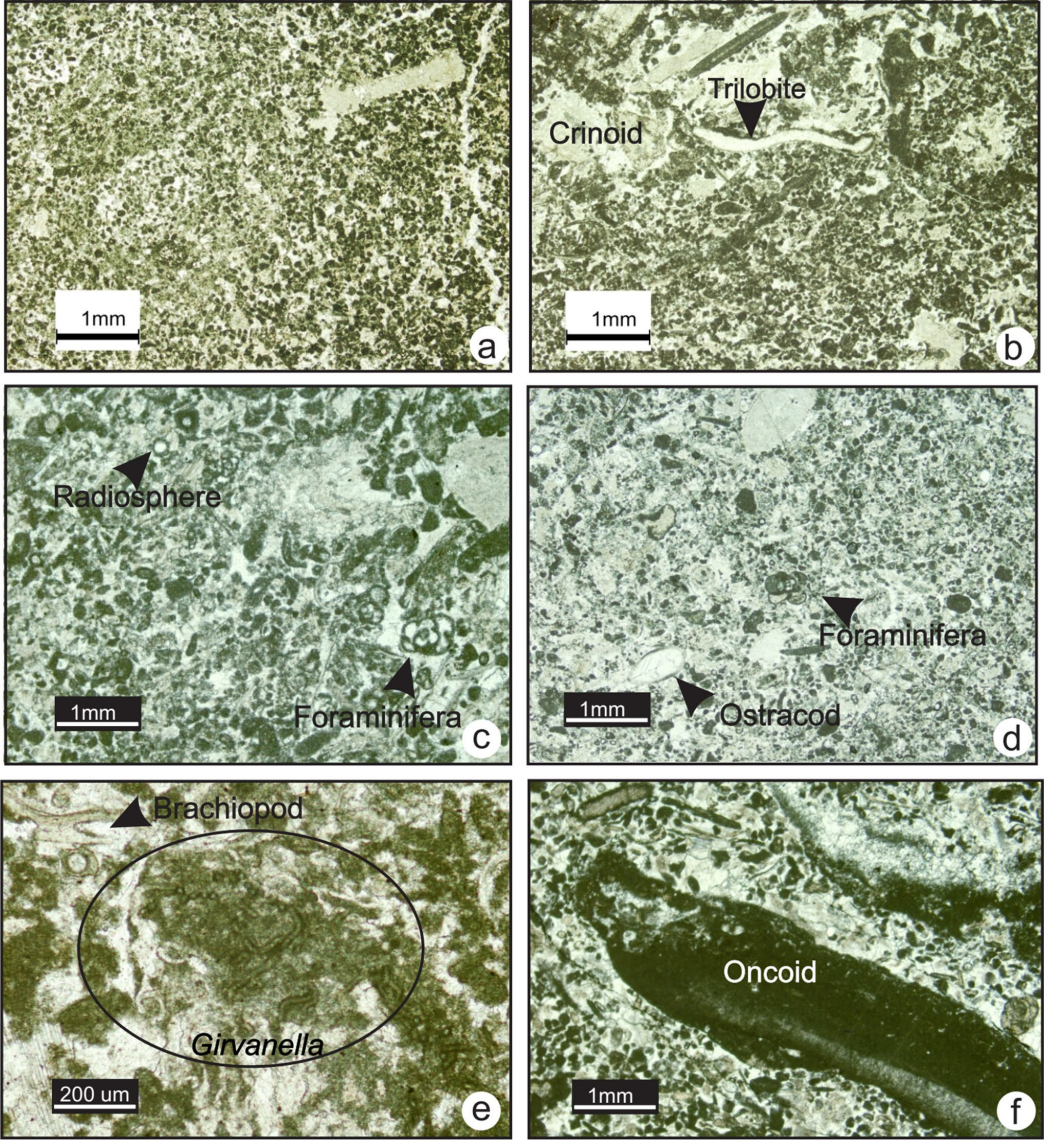
Photomicrographs of MF8. **a** and **b.** Peloidal packstone in the Joana Limestone (TS PH-38 and TS PH-40, section 16). **c** Peloids in the Joana Limestone (TS PH-50, section 16). **d** Benthic fauna in the Joana Limestone (TS BM-102, section 15). **e** *Girvanella* in the Joana Limestone (TS PH-32, section 16). **f** Elongated oncoid (TS BM-92, section 15)

MF7 is an oolitic and bioclastic grainstone with subordinate packstone. This microfacies comprises the most diverse assemblage of skeletal and non-skeletal grains. Ooids are moderately to well sorted, diameters range from 0.2 to 1.5 mm, are well cemented and show little compaction (Fig. 10e and f). Skeletal fragments (calcareous green and red algae, ostracod, echinoderm and bivalve) constitute the nuclei of these ooids. The cortical layers of the ooids are generally well developed with tangential to radial internal fabrics, or both (Fig. 11g). Aggregates of ooids are common and are mainly lumps with well-developed outer micritic cement (Fig. 10g and i). Fibrous marine cement is present around some ooids (Fig. 10g). Other non-skeletal grains consist of rare peloids. Peloids are mostly fully micritized small ooids and other grains. Common to abundant fossil fragments are calcareous green algae, Stacheinaceae, bryozoans, crinoids, echinoids, ostracods, bivalves, brachiopods and endothyrid foraminifera (e.g.* Spinoendothyra* sp., *Latiendothyra* sp. and *Tuberendothyra* sp.) (Fig. 11e and h). Gastropods, trilobite fragments and dasycladacean algae (*Koninckopora* sp.) are rarely observed. Most skeletal grains are rounded and abraded and have non-laminated micritic circum-granular envelopes. In the field, this microfacies occurs in light grey with pale beige weathering, medium to thickly bedded limestone.

This microfacies is interpreted to reflect deposition in open-marine, warm, shallow water (within the upper 20 m of sea level), and above FWWB as shoals and sand bars. In such an environment, the hydrodynamic energy is very high. Active water circulation and warm temperatures promote shallow-marine diagenetic processes including early cementation and micritization.

### MF8-Peloidal packstone

MF8 comprises a photozoan assemblage. It consists of a peloidal packstone with rare to common benthic fossil fragments (Fig. 11a and b). Abundant small peloids and irregular shaped micritized grains are the main grains. Centimetre-sized oncoids and calcified filaments (*Girvanella*) occur in MF8 (Fig. 11e and f). Benthic foraminifera, echinoderm, rare bivalve, trilobite, ostracod and brachiopod fragments are also found (Fig. 11c and d). Calcispheres and radiospheres are rare to common in this microfacies. Thrombolytic-like texture is observed locally. This microfacies is exclusively in the Joana Limestone, southern Nevada.

Oncoids and peloids with foraminifera and benthic fauna fragments point to deposition in a subtidal setting. The subangular shape of the peloids in this microfacies indicates that they may have formed by pervasive micritization of bioclasts leading to the complete loss of the grain’s microstructure. Scarcity of oncoids and lack of spheroidal structures with concentrically stacked laminae in them suggest a low-energy, non-agitated shallow-water environment. This suggests an overall quiet setting but still very shallow and above the FWWB. Association of MF8 with bioclastic and oolitic grainstone (MF7) suggests that this microfacies may have formed in a restricted inner ramp on a back-shoal flank sheltered from wave action.

### MF9-Algal wackestone-packstone

**Fig. 12:**
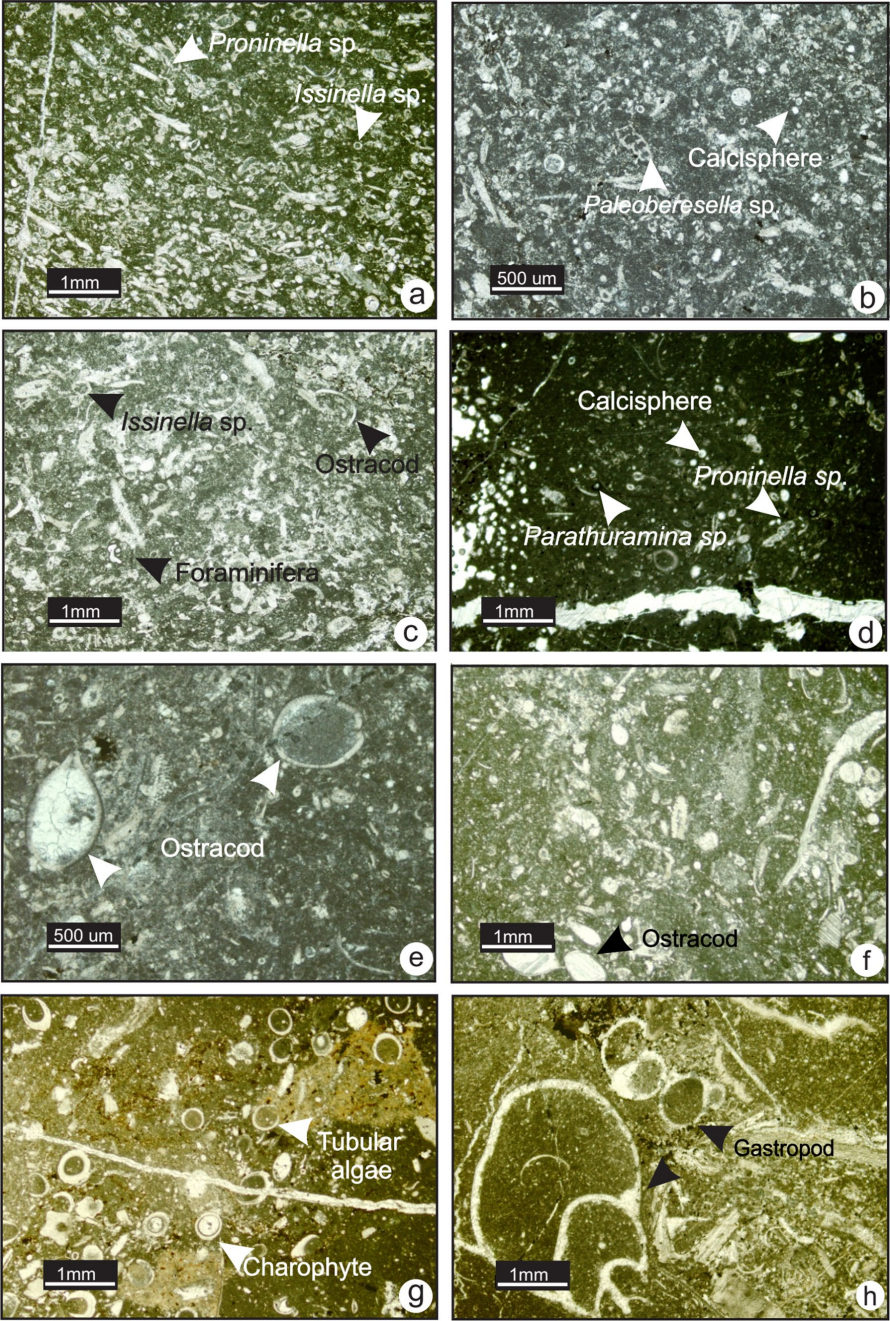
Photomicrographs of MF9 and MF10. **a** Algal packstone in the Palliser Formation (TS P1-12, section 1). **b** Algal packstone in the Joana limestone (BM-39, section 12). **c** Algal fragments, foraminifera and ostracod shells in the Palliser Formation (TS 1–10, section 1). **d** Algal wackestone in the Palliser Formation (TS 1–9, section 1). **e** and **f** Wackestone with intact ostracods in the West Range Limestone (TS BM-42, section 11) and in the Palliser Formation (TS P1-11, section 1). **g** and **h** photomicrographs of MF10. **g** Charophytes wackestone in the West Range Limestone (TS BM-54, section 11). **h** Wackestone with intact gastropods and charophytes in the West Range Limestone (TS BM-54, section 11)

MF9 consists of a wackestone and minor packstone with abundant calcareous green algal fragments, intact ostracods and rare foraminifera (Fig. 12e and f). The green algae observed in this microfacies are *Proninella* sp., *Issinella* sp., *Pseudokamaena* sp. and *Palaeoberesella* sp. Common calcipheres, radiospheres and *Parathuramina* sp. are also found (Fig. 12a to d). The algal fragments are broken but not abraded. The matrix is composed of dense, dark, organic-rich lime mudstone. MF9 is found in association with charophyte algal wackestone (MF10) and fenestral-oncoidal mudstones (MF11). This microfacies is found exclusively in Upper Devonian rocks. MF9 consists of a phototrophic assemblage that requires a light and warm temperature and likely reflects deposition in the proximal shallow subtidal zone of a semi-protected inner ramp.

### MF10-Charophyte algal wackestone (Nevada)

MF10 is a wackestone with abundant charophytes and calcispheres, common intact gastropod and tubular calcareous green algal fragments with rare ostracods (Fig. 12g and h). The matrix is composed of dense, dark, organic-rich lime mudstone. In the field, this microfacies is found exclusively in the West Range Formation in a dark grey, thickly bedded limestone interbedded with coarse sandstone.

The wackestone fabric of MF10 and intact fossils reflect deposition in a moderate to low-energy environment. The biotic assemblage is mostly photozoan that requires light and warm temperature. The occurrence of charophytes and rare presence of a fully-marine fauna such as crinoids and brachiopods suggest fluctuating salinity. This microfacies is consistent with deposition in a brackish-water environment. MF10 is interpreted to reflect deposition in a shallow subtidal to intertidal environment of an inner ramp that was influenced by fresh-water incursions.

### MF11-Peloidal-fenestral mudstone-boundstone

**Fig. 13:**
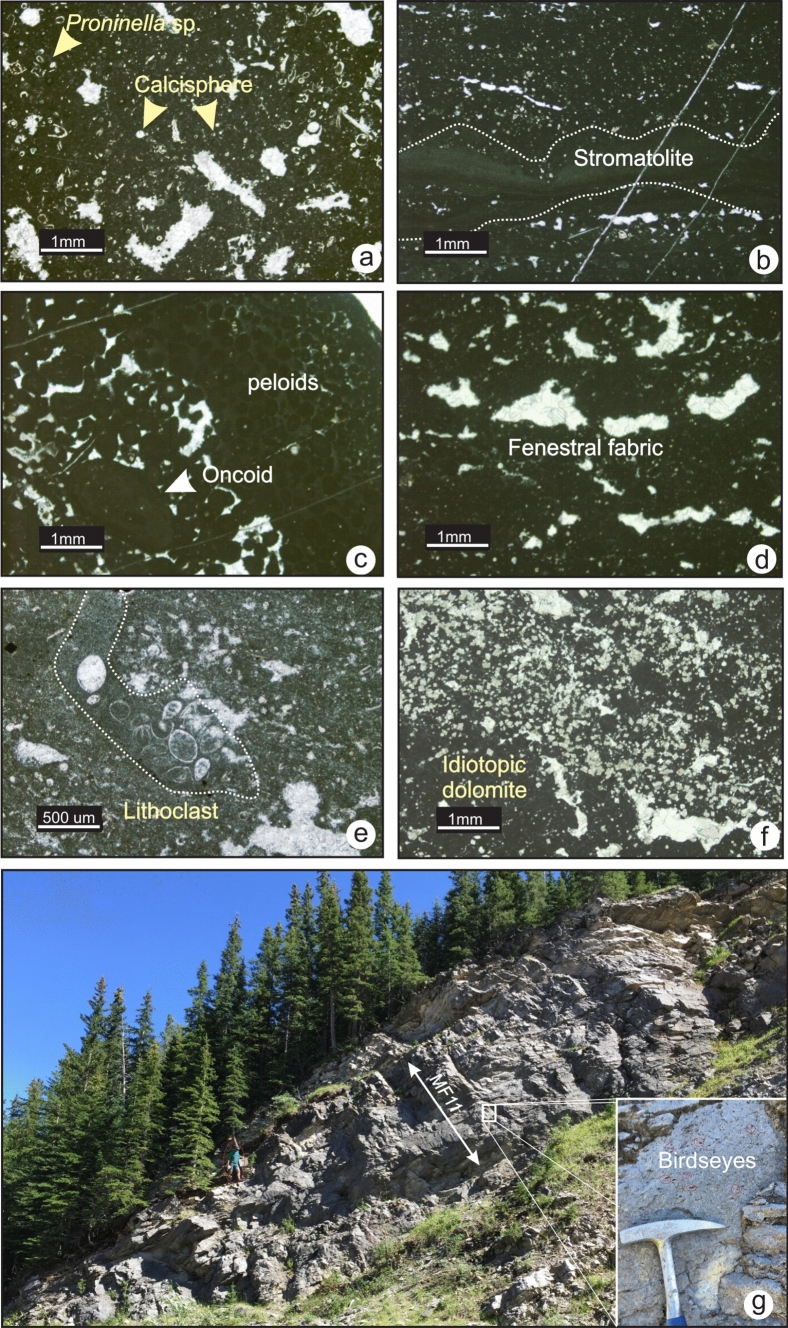
Photomicrographs of MF11. **a** and **b** Fenestral lime mudstone with algal fragments and stromatolitic laminae in the Palliser Formation (TS 1–12 and TS 1–10, section 1). **c** and **d** Peloidal packstone and fenestral fabric in the Shunda Formation (TS 8–92 and TS 8–94, section 6). **e** Ostracod-rich lithoclast (TS 1–9, section 1) in the Palliser Formation. **f** Dolomite crystals in fenestral lime mudstone in the Shunda Formation (TS 8–93, section 6). **g** Fenestral mudstone (MF11) in the Shunda Formation outcrop at Moose Mountain (section 6)

MF11 consists of lime mud-rich boundstone with subordinate peloidal packstone and intraclasts. The prominent feature in this microfacies is the fenestral fabric (Fig. 13a and d). Common cyanobacterial features are stromatolitic laminae and oval-shaped oncoids (Fig. 13b and c). Moderately well-sorted peloids occur (Fig. 13c). Fragments of *Proninella* sp., calcispheres and radiospheres are common (Fig. 13a). Lime mudstone and wackestone lithoclasts, and euhedral (idiotopic) dolomite crystals are also common (Fig. 13e and e). The matrix is muddy and dark. In the field, it occurs in association with LF9 (algal wackestone to packstone) (Fig. 13g). This microfacies is found exclusively in southwestern Alberta within the Palliser and Shunda formations.

Stromatolitic laminae and irregular shaped fenestral fabric with poor lamination are indicative of intertidal to supratidal zones. In such settings, intraclasts originate from the reworking of weakly-lithified carbonate mud or reworking of microbial mats by grazers or desiccation during low tide.

## Oxygen and carbon isotopes

**Fig. 14:**
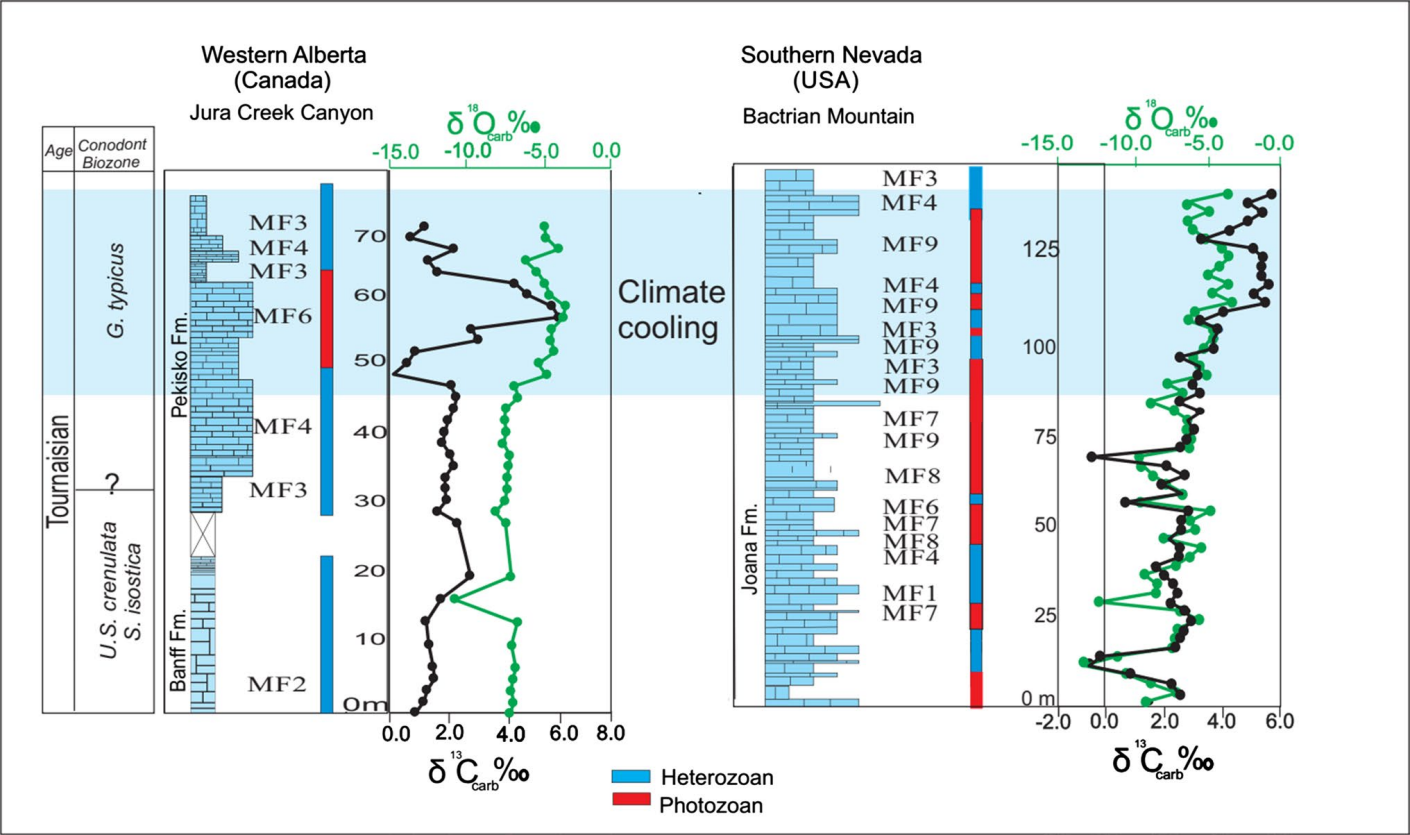
Whole-rock carbon (δ^13^C_carb_) and oxygen (δ^18^O_carb_) isotopes of Tournaisian rocks from Jura Creek, Alberta (Canada) and Bactrian Mountain, Nevada, showing intervals with heterozoan and photozoan assemblages vs isotope excursions

Whole-rock carbonate carbon (*δ*^13^C_carb_) and oxygen isotopes (*δ*^18^O_carb_) were measured in the Banff, Pekisko and Joana formations in Alberta and Nevada. Results from this study show a major positive *δ*^13^C_carb_ excursion in the Tournaisian (*Gnathodus typicus* biozone) with an amplitude of + 4‰ V-PDB in the Pekisko Formation and + 4.1‰ V-PDB in the Joana Limestone. *δ*^18^O_carb_ values show an overall parallelism with trends in *δ*^13^C_carb_ values. Major oxygen isotope excursions coincide with, or are slightly offset from, carbon isotope excursions. A major positive *δ*^18^O_carb_ shift occurs in the Tournaisian (*G. typicus* biozone) with + 2.2‰ in the Pekisko Formation and + 2.2‰ V-PDB in the Joana Formation (Fig. 14). A progressive steady positive δ^13^C_carb_ and δ^18^0_carb_ trend continued throughout the Tournaisian and into the Viséan. [Additional data are given in Online Resource 3 (Isotopic data spreadsheet)].

## Depositional model

### Late Famennian: shallow warm-water carbonate ramp

**Fig. 15:**
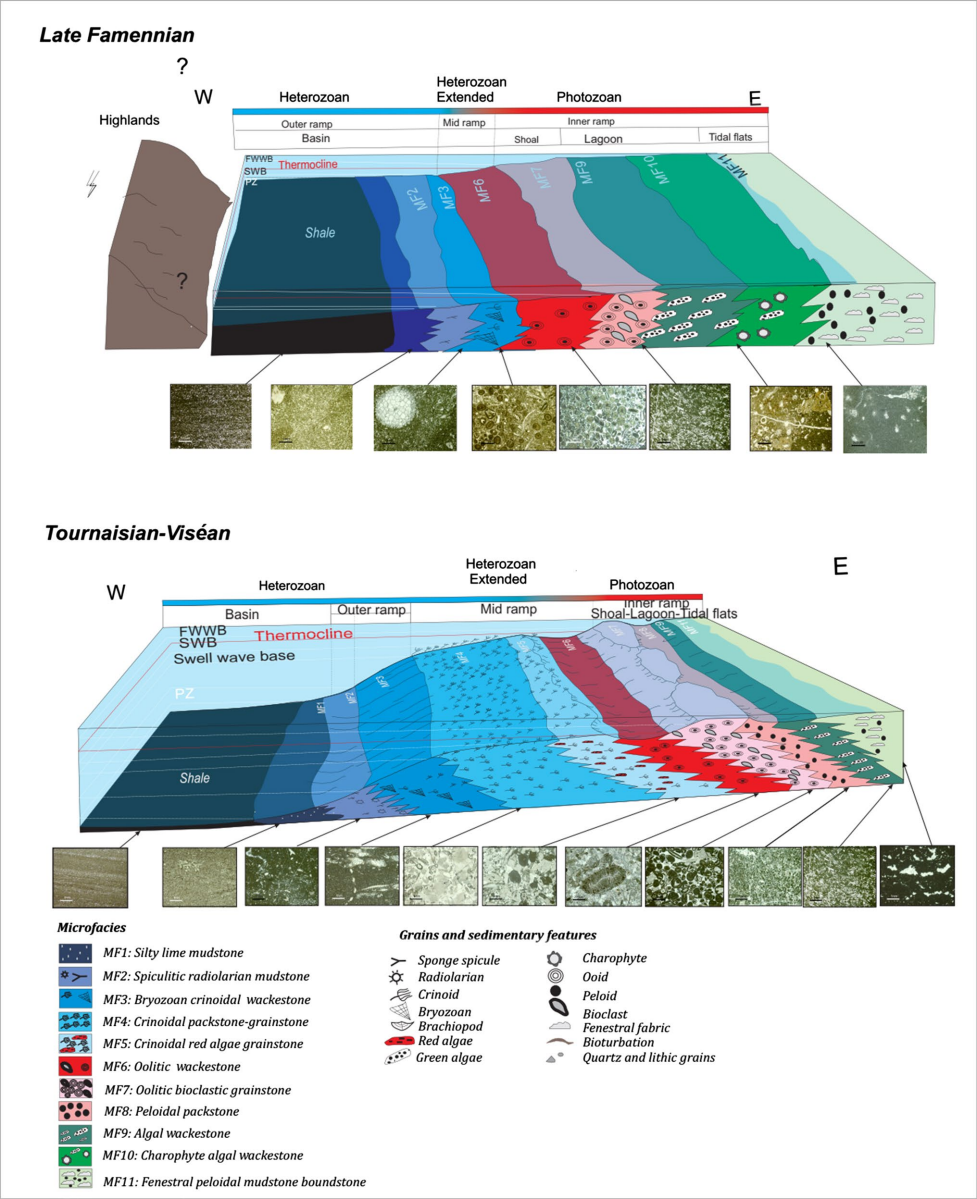
Generalised depositional models for the late Famennian and Tournaisian to Viséan microfacies and lithofacies of Western Laurentia

In the late Famennian, during the time represented by the *P*. *marginifera* to* P*. *expansa* zones, shallow gently dipping carbonate ramps bounded the western margin of Laurentia inboard of an Antler-related geographic barrier to the west (Goebel 1991; Root 2001; Hedhli et al. 2022). The existence of this physical barrier restricted wave energy coming from the open ocean to the shelf (Fig. 15). Warm-water carbonates with photozoan assemblages: oolitic bioclastic grainstone, algal wackestone, charophyte algal wackestone and fenestral peloidal mudstone-boundstone were deposited in the inner ramp (Palliser and West Range formations). Microbial carbonates (microbial laminites, low relief stromatolites and algal wackestone) were prolific indicating mesotrophic conditions, warm temperatures, shallow-water depth, and local brackish areas where charophytes proliferated. Lagoons were rimmed by patchy shoals. These shoals were above the FWWB and might have had little to no effect on the incoming wave energy in the back-ramp area. Cool-water carbonates with a heterozoan assemblage (spiculitic-radiolarian mudstone and bryozoan-crinoidal wackestone) were deposited in the mid and outer ramp areas. A transition zone affected by bathymetric fluctuations of the thermocline existed in the upper mid-ramp where a photozoan-extended assemblage within oolitic wackestone was deposited under low-energy conditions. Grains from shoals were transported and resedimented below the FWWB in a lower-energy environment. Below the thermocline and the SWB crinoidal and bryozoan wackestone was deposited in an area of background sedimentation dominated by lime-mud. Below the PZ, silty lime mudstone and spiculitic lime-mudstone formed in a cool water and in conditions of very little light. Basinward, carbonate sediments graded laterally into laminated black shales (Exshaw, Three Forks, Pilot formations).

### Tournaisian-Viséan: cool-water carbonate factory

During the latest Famennian to earliest Tournaisian in the time represented by the *S*. *praesulcata* to* S*. *quadriplucata* zones, carbonate growth was curtailed by environmental changes and tectonic vicissitudes (Caplan and Bustin 1999; Root 2001; Trexler et al. 2004) causing isolation of carbonate ramps from the open ocean by an Antler related barrier and continental-scale influx of siliciclastic material (Hedhli et al. 2022). In the upper *S*. *quadriplucata* zone a major transgression and deposition of black shale occurred along the western margin of the Laurentia margin (lower Banff Formation in Alberta, upper Sappington Formation in Montana and upper Pilot Shale in Nevada). Carbonate deposition gradually resumed against a background of rapid subsidence and creation of accommodation space (Goebel 1991; Richards et al. 2002). The development of a pronounced slope led to deposition of gravity-assisted deposits (lower to middle Banff and Lodgepole formations). During the Tournaisian to Viséan, a series of distally-steepened carbonate ramps bounded the western margin of the continent (Read 1982) (Fig. 15). The Tournaisian carbonate (Banff, Lodgepole and Joana formations) factories were mainly dominated by heterozoan assemblages. Facies belts that developed below the thermocline covered extensive areas of the ramps relative to the Famennian carbonates, which largely were deposited above the thermocline. During the late Tournaisian to early Viséan in the time represented by the *G*. *crenulata* to* G*. *typicus* zones, warm-water facies developed above the thermocline after progradation of shoals and development of protected back-ramp areas to the west (Pekisko Formation in Alberta, Mission Canyon Formation in Montana, and middle Joana Formation in Nevada). In the basin, black shale was deposited below the PZ and OMZ. In the outer ramp (below the SWB), lime mudstone and spiculitic-radiolarian mudstone to wackestone were deposited in the lower to mid slope environments. Bryozoan and crinoidal wackestones were deposited below the SWB in the upper slope. The outer ramp setting is overall low energy, allowing accumulation of mud-sized material. However, during storms localized packstones accumulated. The mid ramp shows the greatest changes during the Early Mississippian compared to Devonian ramps. Crinoids colonized the seafloor forming extensive crinoidal banks in a moderate to high-energy environment that did not promote deposition of lime-mud. Crinoidal grainstone and packstone were deposited on the mid ramp in areas affected by the occasional storm but more often by swell waves. Red algae and foraminifera dominated these slightly shallower, cool-water environments (Mamet 1977). The relief and spatial distribution of the crinoidal banks led to attenuation of swell wave energy creating a low-energy environment behind the crinoidal banks where crinoidal wackestones accumulated with resedimented photozoan bioclasts derived from nearby shoals. Oolitic and bioclastic shoals accumulated in the inner ramp above the thermocline and FWWB base. Behind these shoals, the warm-water temperature and low-energy led to accumulation of peloidal packstone and algal wackestone in a lagoonal setting similar to the Devonian facies of the Palliser Formation. In the most proximal setting tidal flats existed where fenestral and peloidal boundstone and mudstone accumulated following development and decay of microbial mats.

## Discussion

### Famennian: low accumulation rates and lime-mud abundance

Famennian carbonate ramps such as the Palliser and West Range developed in the aftermath of the Frasnian-Famennian mass extinction, and during a time when reef communities collapsed (Copper 2002; Webb 2002). Carbonate accumulation rates were low (approximately 20–50 m Myr^−1^) (Peterhänsel et al. 2008). The overall architecture of these ramps was assumed to be monotonous and “layer-cake” based on the extensive nature of individual depositional areas and parallel reflectors in subsurface seismic profile, e.g., in the Wabamun Formation, the Palliser Formation correlative in Western Canada (Peterhänsel et al. 2008). However, this study and the Peterhänsel et al. (2008) detailed study on the Palliser Formation revealed hitherto unrecognized low-angle facies diachroneity. Overall, the latest Famennian carbonate factories comprised mud- and peloid-producing and precipitating micro- and macro-organisms: green calcareous algae, crinoids and to a lesser degree brachiopods, gastropods, ostracods and stromatoporoids. The benthos was mainly a mesotrophic community (Peterhänsel et al. 2008). The monotonous appearance of these carbonates is due to their richness in lime mud which resulted from active micritization of skeletal grains and bioclasts, bioerosion and breakdown of bioclasts before burial (Peterhänsel and Pratt 2001), calcification of cyanobacteria, accumulation of faecal pellets (100 µm in diameter peloid), microbially-induced cements (50 µm in diameter automicritic peloids; Chafetz 1986), and accumulation of steinkerns (100–200 µm in diameter algal tubes filled with lime mud) (Fig. 14a to e). The Famennian carbonate ramps were also marked by heavily burrowed firm-grounds particularly present in the upper part of the Palliser Formation. These firm-grounds are indicative of periods of decline in carbonate accumulation and enhanced early carbonate cementation (Goldring 1995). The pervasive bioturbation of these surfaces is suggested to be the artifact of organisms belonging to the fodinichnia-producing crustaceans (Myrow 1995). These organisms create dense networks of open burrows down to several decimetre in depth that are subsequently filled with coarse sediments during storm events (Wanless et al. 1988).

### Tournaisian-Viséan: high accumulation rates and lack of lime mud

The lower Mississippian carbonate has a distally-steepened ramp architecture deposited in a temperature-stratified water column that separated photozoan from heterozoan assemblages (Brandley and Krause 1997; Martindale and Boreen 1997) similar to the modern Lincoln shelf of the southern coast of Australia (James 1997). Mississippian carbonate ramps developed in the aftermath of the Hangenberg faunal crisis (Walliser 1984) and were also marked by constant carbonate production over much of the ramp, in the absence of reef builders (Wright and Faulkner 1990). However, the thickness of crinoidal facies belts, notably in the Banff, Lodgepole and Joana formations, attests to high accumulation rates over less than 4 My in the early Tournaisian, from lower *S*. *crenulata to G. typicus* zones. High sedimentation rates are uncharacteristic of cool-water carbonate factories (James 1997). Modern cool-water carbonates have low accumulation rates; for instance, the Lincoln carbonate shelf is a large ramp with thin metre-scale inner to mid ramp sedimentary packages (Boreen and James 1993; James et al. 1994). High accumulation rates of Mississippian carbonates might be partially explained by high subsidence rate allowing faster burial and preservation and ecological changes. One common aspect of Mississippian carbonates is their richness in crinoids. In western Canada and western USA, crinoidal grainstone cliffs of the Banff, Lodgepole and Joana formations reach several hundreds of metres in thickness. Numerous workers have suggested that this peak richness in crinoids was primarily a function of Early Carboniferous originations rather than expansion of Devonian holdover taxa (Ausich and Kammer 2013). They also proposed that origination was higher in North America than anywhere else (Sepekoski 1996; Ausich and Kammer 2013). Two factors are behind crinoids reaching their Phanerozoic peak of generic richness during the Early Carboniferous. The first factor is the expansion of Tournaisian carbonate ramps following the Frasnian mass extinction of reef faunas. The second factor is the Tournaisian surge of predatory durophagous fish following the Hangenberg extinction (Ausich and Kammer 2013). Throughout the Carboniferous, gradual reduction in the area of carbonate ramps coupled with radiation of new durophagous fish led to a continuous background extinction of crinoids without any obvious ecological replacement (Ausich and Kammer 2013). Another prominent feature of Mississippian carbonates is their lack of lime mud in the mid-to-outer ramp sub-environments. A plausible explanation is the cool-water temperatures that inhibited microbial and other organisms producing lime mud. Additionally, although the ramp seems to be subject to high-energy winnowing as reflected by thick packages of crinoidal tempestites, high sedimentation rates might explain the lack of bioerosion and mechanical erosion to breakdown clasts into finer lime-mud size. Residence-time of the bioclasts on the seafloor was brief before their rapid burial during subsequent storm events.

### Cause(s) of cooling and carbonate factory turnover

#### Global cooling

Deposition of upper Devonian to Mississippian carbonates of western Laurentia coincided with the transition from the Devonian greenhouse to the Carboniferous icehouse (Frakes et al. 1992; Montañez and Poulsen 2013). Occurrence of glacial deposits in Bolivia, Brazil and Peru indicates that the icehouse might have started as early as the late Famennian as a series of short-lived glacial events in Gondwana (Isaacson et al. 2008). Carbonate depositional changes might also be a record of the complex responses of atmospheric and oceanic circulation, CO_2_ levels, and lower-latitude climate due to the waxing and waning of the Gondwana ice sheet (e.g. Birgenheier et al. 2010). The Tournaisian positive excursions in *δ*^18^O_carb_ (+2.2‰ in the Pekisko Formation and  +2.2‰ V-PDB in the Joana Formation) and *δ*^13^C_carb_ (+4‰ V-PDB in the Pekisko Formation and  +4.1‰ V-PDB in the Joana Formation) isotopes during the *G. typicus *zone (Fig. 14) might coincide with the first prominent pulses of the Carboniferous glaciations. While the *δ*^13^C_carb_ positive excursion might reflect the Late Famennian to early Tournaisian withdrawal and burial of light carbon isotopes in organic-rich black shales caused by a lack of recycling of organic matter (Tyson and Pearson 1991; Cheng et al. 2020), positive shifts in *δ*^18^O_carb_ could correspond to the formation of ice caps in southern Gondwana. Oxygen isotope ratios of whole rock including cement phases is known to be susceptible to diagenetic alteration (Marshall 1992). To evaluate sea-water temperatures recent studies have used oxygen isotope ratios of well-preserved calcitic brachiopod shells or of conodont apatite known as reliable palaeotemperature proxies due to these fossils’ resilience to diagenetic alteration (e.g. Veizer et al. 1999; Buggisch et al. 2008). Although whole-rock oxygen isotopes were used in this study, our results agree with Buggisch et al. (2008) data from the Pekisko Formation in western Canada. Buggisch et al. (2008) showed a major positive excursion in δ^13^C_carb_ (up to  +6.5‰ V-PDB) and δ^18^O _apatite_ (+1.5‰ V-SMOW) from sections in Europe and Canada in the *G. typicus* zone and suggested that these excursions correspond to the first major cooling and potential glaciation event that occurred in the Tournaisian with ice masses persisting into the Viséan. This study has documented the occurrences of warm-water carbonate facies in the Pekisko, Mission Canyon and Joana formations, namely oolitic and bioclastic grainstone during the proposed peak of cooling (Buggisch et al. 2008). Temperature stratification in the water column with warm surface waters is unlikely to have occurred under a cool climate. This suggests that locally in western Laurentia the ocean and the atmosphere were likely decoupled with warm-water marine temperatures occurring in shallow-water environments. Thus, the more plausible explanation to oceanographic cooling and carbonate turn-over in western Laurentia is the occurrence of cold/cool-water upwelling causing bathymetric thermocline fluctuation rather than climate cooling (Brandley and Krause 1997; Martindale and Boreen 1997). A similar scenario is suggested for the Pleistocene Ice Age, where warm surface-water temperatures persisted in the eastern Atlantic (Schefuß et al. 2004). Other alternative, geochemical excursions of this study and Buggisch et al. (2008) do not correspond to climate cooling, but rather to diagenetic alteration signals or other events. In the latter case, the upwelling hypothesis also provides a plausible explanation to the oceanographic cooling along western Laurentia at low latitudes.

#### Upwelling

The palaeogeographic position of lower Mississippian carbonate ramps, facing the open eastern proto-Panthalassa ocean at low latitudes (0–20° N) and exposed to the eastern boundary current of the tropical region, would have driven coastal upwelling. Upwelling of cold waters would have led to shoaling of the thermocline and promoted growth of a heterotrophic biota over large areas of the ramps. With the end of the Upper Devonian to Lower Mississippian pulse of the Antler Orogeny, oceanographic changes took place (Root 2001; Hedhli et al. 2022). Wind-driven upwelling of cold waters (Ekman transport) occurred on the newly open west coast (Alberta, Montana), similar to present-day California. Upwelling of cold waters led to shoaling of the thermocline and eastward displacement of warm-water facies. Under cool temperature and high energy (wave-dominated coast) the thriving heterotrophic sessile carbonate secreting crinoids, bryozoans and red algae were buried and preserved as crinoidal grainstones. Long-shore currents causing invasion of cool water from the north might have led to a marine water-cooling down to southwestern Laurentia (Nevada) and accumulation of cool-water facies of the upper parts of the Joana Limestone. Phosphate enrichment of uppermost Devonian carbonates in western Canada supports the upwelling hypothesis as the main driver of carbonate turnover (Li et al. 2022).

## Conclusion

In this study, the Upper Devonian to Lower Mississippian sedimentary succession extending from western Canada to southern Nevada is revisited to examine the turnover of Late Devonian tropical warm-water carbonate ramps to Early Mississippian cool-water carbonate factories. Eleven carbonate (11) microfacies were identified and their spatial and temporal distribution used to propose new depositional models for the Late Devonian and Early Carboniferous. Late Devonian shallow warm-water photozoan dominated, and highly mesotrophic carbonate ramps were succeeded by Early Mississippian cool-water heterozoan-dominated distally-steepened carbonate ramps with high accumulation rates. Mississippian carbonates along the western margin of Laurentia record positive excursions of whole-rock carbon and oxygen isotopes, which likely correspond to the first cooling peak during the transition to the Carboniferous-Permian icehouse climatic mode. However, persistence of localized warm-water facies indicates that climate cooling was not the main driver for the thermocline shoaling. During the earliest Carboniferous, shoaling of the thermocline is most likely a result of plate reorganization and cold-water upwelling along an open coast, as the Late Devonian Antler orogen no longer provided an oceanic obstruction to the west.

## Supplementary Information

Below is the link to the electronic supplementary material.

Supplementary file1 (PDF 954 KB) Online resource 1 include the methods used for sampling, sedimentology and isotope geochemistry

Supplementary file2 (XLSX 19 KB) Online resource 2 includes examples of stratigraphic columns with microfacies trends

Supplementary file3 (XLSX 19 KB) Online resource 3 includes carbon and oxygen isotopes data
