# Supplementary material for: Devonian to Carboniferous continental-scale carbonate turnover in Western Laurentia (North America): upwelling or climate cooling?
Source: Facies. 2022 Jul 25;68(4):15. doi: 10.1007/s10347-022-00653-4 (PMC9314305; doi:10.1007/s10347-022-00653-4)
Supplement: Supplementary file 1 — Supplementary file1 (PDF 954 KB) Online resource 1 include the methods used for sampling, sedimentology and isotope geochemistry [file 10347_2022_653_MOESM1_ESM.pdf]

DEVONIAN TO CARBONIFEROUS CONTINENTAL SCALE CARBONATE TURNOVER IN WESTERN LAURENTIA (NORTH AMERICA): UPWELLING OR CLIMATE COOLING?

Makram Hedhli [1,2], Keith Dewing [1], Benoit Beauchamp [2], Stephen E. Grasby [1,2], Rudi Meyer [2]

[1] *Geological Survey of Canada, 3303-33rd Street NW, Calgary, Alberta T2L 2A7, Canada*

[2] *University of Calgary, Department of Geoscience, 2500 University Drive NW, Calgary, Alberta, T2N 1N4, Canada*

Corresponding Author: *Makram Hedhli**; email:*** *Makram.Hedhli@RNCan-NRCan.gc.ca*

METHODS

Sampling

Sections were measured from the uppermost beds of the Devonian, through the DC boundary, into the Mississippian carbonates. Samples were collected every 10–30 cm across sharp stratigraphic contacts, but at larger intervals (1.5–3m) when lithology seemed uniform. In Alberta, most sections were measured from the uppermost beds of the Palliser Formation, through the Exshaw and Banff formations, into the overlying Pekisko, Livingstone and Shunda formations. In southwestern Montana, outcrops were sampled from the Devonian Three Forks Formation through the Sappington Formation, and into the Mississippian Lodgepole and Mission Canyon formations. In Nevada, the sampling was conducted from the Devonian West Range Formation into the Mississippian Joana Limestone. Thin sections were prepared from samples from these sections. Microscopy of thin sections was done at the University of Calgary using a Nikon Eclipse LV100 POL petrographic microscope equipped with a Nikon digital camera

(DXM1200C) and ACT-1 software was used to examine thin sections and to produce photographs.

Sedimentology

Microfacies analysis is a tool to classify carbonate rocks, interpret depositional environments and diagenetic histories based on grain size, grain composition, cements, fossils and textures. Carbonate rocks (>70% carbonate material) and mixed siliciclastic carbonate (30–70% carbonate material) samples were classified according to Dunham (1962) carbonate classification scheme. Fossil assemblages were quantified using four divisions based on qualitative abundance: dominant (D) allochem is the most abundant allochem throughout the thin section and has high number of occurrences within a single field of view in 2x objective lens relative to other allochem; abundant (A) allochem is visible throughout the thin section and has multiple occurrences within a single field of view in 2x objective lens; common (C), allochem is present in most field of views with a 2x objective lens; and rare (R), two or fewer specimens throughout the slide. Siliciclastic (<10% carbonate material) and calcareous siliciclastic (10–30% carbonate material) rocks were classified based on grain size and composition. The terms photozoan, heterozoan-extended, and heterozoan refer to fossil assemblages that reflect the relative dominance of phototrophic versus heterotrophic biota. Photozoan sediments only develop in warm, illuminated, shallow neritic environments. Heterozoan sediments can form in warm and cool marine environments at any depths (James, 1997). The term heterozoan-extended is applied to mixing of heterozoan assemblage with certain photozoan elements.

Stable isotope Geochemistry

Powdered fresh carbonate samples of bulk rock (i.e. fossils, micrite and various cement phases), including duplicates, were analyzed for $\delta^{13}C_{VPDB}$ and $\delta^{18}O_{VPDB}$ values of carbonates at the Isotope Science Laboratory at the University of Calgary. During sample processing, weathered surfaces were removed, and fresh samples were powdered. Approximately 2 mg of powdered rock sample were reacted with anhydrous phosphoric acid in a test tube at 25 °C. The evolved CO_2 was cryogenically distilled and analyzed for $^{13}C/^{12}C$ and $^{18}O/^{16}O$ ratios with a VG 903 ratio mass spectrometer. To normalize the data and correct for any instrument errors, selected internal standards were run at the beginning and end of the sample set. The results are expressed as δ-values relative to the Vienna Peedee Belemnite standard (V-PDB). The precision and accuracy of the $\delta^{13}C_{VPDB}$ and $\delta^{18}O_{VPDB}$ analysis is 0.2‰.
